# Synergistic dual cell therapy for atherosclerosis regression: ROS-responsive Bio-liposomes co-loaded with Geniposide and Emodin

**DOI:** 10.1186/s12951-024-02389-5

**Published:** 2024-03-25

**Authors:** Zhenxian Li, Haimei Zhu, Hao Liu, Dayue Liu, Jianhe Liu, Yi Zhang, Zhang Qin, Yijia Xu, Yuan Peng, Lihua Ruan, Jintao Li, Yao He, Bin Liu, Yun Long

**Affiliations:** 1 Department of Cardiology, The First Hospital of Hunan University of Chinese Medicine, Changsha, 410007 China; 2 College of Biology, Hunan University, Changsha, 410082 China; 3 Department of Rehabilitation, The Second Xiangya Hospital, Central South University, Changsha, 410011 China; 4 NHC Key Laboratory of Metabolic Cardiovascular Diseases Research, Ningxia Medical University, Yinchuan, 750004 China; 5 Department of Pain, The First Hospital of Hunan University of Chinese Medicine, Changsha, 410007 China

**Keywords:** Atherosclerosis, ROS-responsive, Biomimetic liposome, Geniposide, Emodin

## Abstract

The development of nanomaterials for delivering natural compounds has emerged as a promising approach for atherosclerosis therapy. However, premature drug release remains a challenge. Here, we present a ROS-responsive biomimetic nanocomplex co-loaded with Geniposide (GP) and Emodin (EM) in nanoliposome particles (LP NPs) for targeted atherosclerosis therapy. The nanocomplex, hybridized with the macrophage membrane (Møm), effectively evades immune system clearance and targets atherosclerotic plaques. A modified thioketal (TK) system responds to ROS-rich plaque regions, triggering controlled drug release. In vitro, the nanocomplex inhibits endothelial cell apoptosis and macrophage lipid accumulation, restores endothelial cell function, and promotes cholesterol effluxion. In vivo, it targets ROS-rich atherosclerotic plaques, reducing plaque area ROS levels and restoring endothelial cell function, consequently promoting cholesterol outflow. Our study demonstrates that ROS-responsive biomimetic nanocomplexes co-delivering GP and EM exert a synergistic effect against endothelial cell apoptosis and lipid deposition in macrophages, offering a promising dual-cell therapy modality for atherosclerosis regression.

**Supplementary Information:**

The online version contains supplementary material available at 10.1186/s12951-024-02389-5.

## Highlights

The first report on ROS-responsive biomimetic nano complex co-loaded with Geniposide and Emodin for targeted atherosclerosis therapy.

Discovery of the synergistic effect of simultaneous administration of Geniposide and Emodin in inhibiting endothelial cell apoptosis and macrophage lipid deposition, key steps in plaque progression.

Utilization of macrophage membrane (Møm) hybrid nanocomplex to avoid clearance by the immune system and enhance targeting ability.

Development of a modified thioketal (TK) system that responds to ROS-rich plaque regions, enabling accurate and rapid drug release.

In vitro and in vivo evidence demonstrating the efficacy of ROS-responsive biomimetic nanocomplexes in reducing plaque area ROS levels, restoring endothelial cell function and reducing lipid deposition, with potential implications for atherosclerosis treatment.

**Supplementary Information:**

The online version contains supplementary material available at 10.1186/s12951-024-02389-5.

## Introduction

Atherosclerosis (AS) is a chronic, multifactorial disease that underlies many life-threatening cardiovascular events [1–3]. The development of atherosclerosis involves key processes such as endothelial cell apoptosis and lipid deposition in macrophages [4, 5]. Endothelial cell apoptosis is believed to be an early event in the development of atherosclerosis. A large number of endothelial cells undergo apoptosis, leading to endothelial dysfunction and the formation and progression of atherosclerotic lesions [6, 7]. Furthermore, the damage to the endothelial barrier and the enlargement of gaps allows circulating lipoprotein particles to infiltrate the vessel wall. The lipoprotein particles are then modified and phagocytosed by macrophages, resulting in the formation of foam cells and the deposition of lipids, thus promoting the progression of atherosclerosis [8]. Therefore, finding solutions to address endothelial cell apoptosis and macrophage lipid deposition simultaneously holds promise for the effective management of atherosclerosis.

Geniposide (GP), the main active ingredient of *Gardenia jasminoides Ellis*, a Chinese medicinal herb, has been found to inhibit apoptosis of human umbilical vein endothelial cells (HUVECs) and protect against endothelial damage [9, 10]. Study has shown that the administration of GP significantly improves endothelial function, which is helpful for AS treatment [11]. Moreover, this compound with antioxidant, anti-inflammatory, antithrombotic function can inhibit smooth muscle cells (SMCs) proliferation and migration, regulate polarization of macrophages, and inhibit lipid deposition [10, 12–14]. Emodin (EM), an important active component of *Rhubarb*, has been reported to promote cholesterol outflow from macrophages and reduce lipid accumulation [15]. Treatment with Emodin has also been shown to effectively improve the serum lipid profile in rats fed a high-cholesterol diet [16]. In addition, EM also showed anti-inflammatory, antioxidant, anti-platelet function and regulate polarization of macrophages [17–20]. Given these individual benefits, combining GP with EM is expected to have synergistic effects on preventing plaque progression by regulating apoptosis and lipid accumulation. However, the limited oral availability and susceptibility to liver first-pass effect currently limit the application of this combination therapy.

Nanoengineering technology has been used to treat a variety of cardiovascular diseases [21, 22]. Based on our previous research, we have found that nano-liposomes (LP NPs) are a promising approach for treating AS [23]. Using macrophage cell membranes (Møm) to disguise nanoparticles is an established treatment [24], it can improve the body’s elimination of NPs and prolong half-life in the bloodstream. Additionally, this method allows for targeted delivery of the drug to the AS plaque site due to the interaction of receptors on the surface of damaged endothelial cells with macrophage ligands [25, 26]. This targeted approach holds great potential for effectively treating AS. Moreover, there is increasing evidence suggesting that excessive reactive oxygen species (ROS) caused by mitochondrial dysfunction play a role in endothelial apoptosis and lipid accumulation, ultimately leading to the development of AS [27]. Utilizing the overexpression of ROS as a trigger, we can achieve accurate and efficient drug release, specifically at atherosclerotic sites. The modification of a ROS-responsive linker, such as thioketal (TK), enables the “smart” release of drugs and precise delivery to the targeted area [28, 29]. This approach ensures targeted drug delivery in a controlled manner. Therefore, combining LP NPs with macrophage cell membrane encapsulation and ROS-responsive drug release has the potential to revolutionize AS treatment by improving drug targeting and efficacy.

In this study, we have developed a ROS-responsive biomimetic nanodrug delivery system for the treatment of AS. Our system involves loading the anti-apoptosis drug Geniposide (GP) and lipid-removal drug Emodin (EM) together into LP NPs and modifying them with a ROS-sensitive linker called thioketal (DSPE-TK-PEG_2000_). To further enhance the circulation half-life and targeting ability of the nanomedical drug, we hybridized macrophage cell membrane (Møm) onto the surface of the nanoparticles, resulting in TK-MLP@(GP + EM) NPs. TK-MLP@(GP + EM) NPs aims to prevent AS through the following mechanisms: (1) The hybridization of macrophage membrane enables long circulation and efficient targeting. (2) The high ROS levels in AS plaques serves as an intelligent “drug release switch”. When the DSPE-TK-PEG_2000_ linker is broken in response to ROS, the structure of the nanoparticles is destroyed, thereby releasing the drugs from the LP NPs. (3) By simultaneously achieving simultaneous anti-endothelial apoptosis and lipid-removal synergistic therapy, our system holds the promise of effectively treating atherosclerosis (Scheme). Overall, our proposed nanomedicine shows great potential for the treatment of atherosclerosis by addressing multiple aspects of the disease.

**Scheme:**
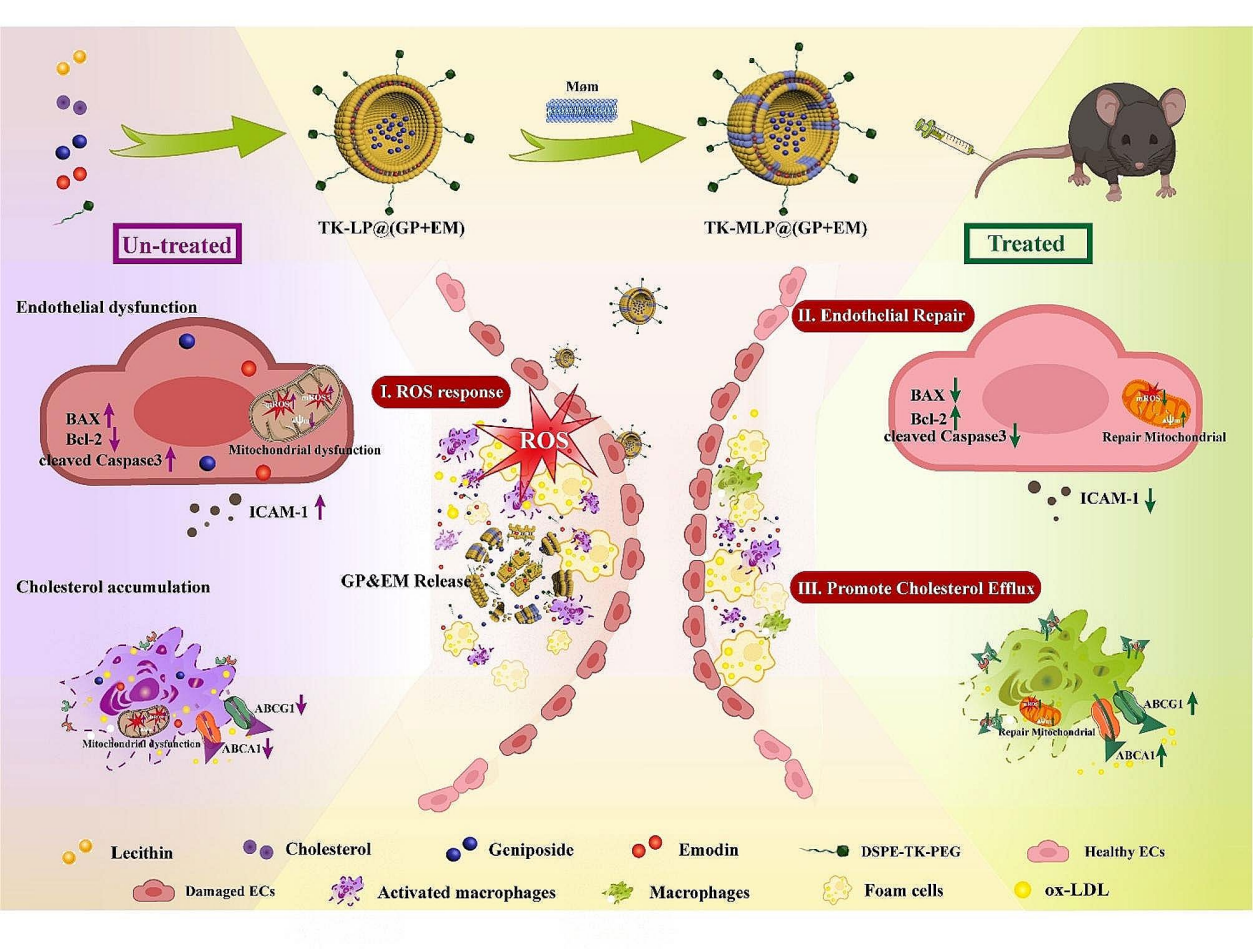
Schematic preparation of TK-MLP@(GP + EM) NPs and the strategy for atherosclerosis treatment. Firstly, nanoliposomes (LP NPs) were utilized as carriers for co-loading Geniposide (GP) and Emodin (EM) with a high drug-loading efficiency. Next, a ROS-sensitive linker known as DSPE-TK-PEG_2000_ was employed to modify the nanoliposomes. Finally, the nanoliposomes were masked with the macrophage cell membrane (Møm), resulting in the formation of TK-MLP@(GP + EM) NPs. Upon intravenous administration, the TK-MLP@(GP + EM) NPs exert their inhibitory effect on AS through a well-defined mechanism. Specifically, large amounts of ROS produced by dysfunctional mitochondria can trigger the structural destruction of the nanosystem, with rapid release of GP and EM, leading to the inhibition of endothelial apoptosis as well as the reduction in macrophage lipid accumulation, both of which are critical steps involved in the progression of plaques. Therefore, the TK-MLP@(GP + EM) NPs is effective in the treatment of AS.

## Experimental section

### Materials and reagents

Emodin was obtained from Chenguang Biotechnology Co., LTD (Xi’an, China). Geniposide was purchased from Shanghai Macklin Biochemical Co., Ltd (Shanghai, China). L-alpha-phosphatidyl choline (SPC) and cholesterol were acquired from Shanghai Ryon Biological Technology Co. Ltd (Shanghai, China). DSPE-TK-PEG_2000_ was obtained from Xi’an RuiXi Biological Technology Co., LTD (Xi’an, China). Additionally, 2′,7′-Dichlorofluorescin diacetate was purchased from Beijing Solarbio Science & Technology Co., Ltd. (Beijing, China), MitoSox Red was obtained from MCE (MedChemExpress), and MitoQ was acquired from GLPBIO. Antibodies, including VE-cadherin, cleaved caspase-3, BAX, Bcl-2, and β-actin, were purchased from Proteintech (IL, USA).

### Preparation of macrophages membrane (Møm)

To prepare the macrophage membrane (Møm), RAW264.7 cells were isolated as previously described [23]. Briefly, the cells were dispersed in reagent A (Beyotime, China) containing 1 mM PMSF and cooled to 4 °C for 30 min. Ultrasound (80 W) was then used to break the cell suspension for 10 min. The mixture underwent four cycles of freezing and thawing at -80 °C and 37 °C. Afterward, the mixture was centrifuged at 800 rpm for 10 min. The resulting supernatant was subjected to an additional centrifugation at 13,000 rpm for 30 min, yielding Møm in the precipitates. The Møm was stored at -80 °C for future use.

### Preparation of TK-MLP@(GP + EM) NPs

The production of TK-LP@(GP + EM) NPs was carried out using the thin film hydration method, as previously reported [30]. In this process, 20 mg of soybean lecithin, 10 mg of cholesterol, and 6 mg of DSPE-TK-PEG_2000_ were dissolved in 5 mL of CHCl_3_. A solution of 1 mg/mL Emodin was added to this mixture. The solution was then transferred to a round-bottomed flask, and the organic solvent was evaporated using a rotary evaporator, forming a lipid film at the bottom of the flask.

Next, 3 mL of a PBS solution containing 2% Tween-80, 0.5% MethylCellulose and Geniposide (3 mg) and was added to the flask to hydrate the lipid film. The mixture was sonicated on ice for 10 min. Subsequently, the emulsion nanoliposome suspension was obtained by passing it through a 0.45 μm microporous filtration membrane, effectively removing free Emodin and Geniposide through centrifugation at 13,000 rpm for 30 min.

The resulting TK-LP@(GP + EM) NPs were then mixed with the Møm solution according to the ratio of lecithin (Møm: liposome = 1:10(w/w)), and the mixture was stirred at 37 °C for 2 h. The uniform TK-MLP@(GP + EM) NPs were obtained by sonication in a water bath and extrusion through a 0.22 μm polyethersulfone membrane.

### Characterization

Transmission electron microscopy (TEM) was used to observe the topographical characteristics of the nanoparticles. Furthermore, the morphologies of TK-MLP@(GP + EM) NPs were imaged by TEM after treatment with 1 mM H_2_O_2_ for 4 h [28]. Dynamic light scattering (DLS) using a Nano ZS90 Zetasizer (Malvern) was employed to determine the size and zeta potential of the nanoparticles. A membrane protein extraction kit was used to extract the macrophage membrane and the [LP+Mø]m NPs for protein extraction. The extracted protein samples were separated by 8% SDS-PAGE gel and stained with Coomassie bright blue. Förster resonance energy transfer (FRET) was used to characterize the fusion efficiency between LP NPs with Møm. Briefly, DiI (Ex/Em = 549/565 nm) and DiD (Ex/Em = 644/663 nm) were added to the LP NPs and stirred at 37℃ for 1 h, centrifuged at 13,000 rpm for 15 min, then washed away the free dye. Subsequently, Møm was added to the DiI/DiD-dyed LP NPs and stirred at 37℃ for 2 h to promote fusion. We used LP NPs without Møm was used as a control. The fusion of LP NPs with Møm was reflected by detecting the donor’s recovery fluorescence (DiI), with the detection parameters of Ex = 525 nm and Em range of 550 ∼ 750 nm.

To investigate the release behaviors of GP and EM from TK-MLP@(GP + EM) NPs at pH 7.4, the nanoparticles were placed in dialysis bags with a molecular weight cutoff of 3,500 Da. The bags were immersed in 50 mL of 1 mM H_2_O_2_ (pH 7.4) and stirred in a 37 °C water bath. At pre-determined time points, 1 mL of the solution was sampled and replaced with an equal volume of H_2_O_2_ solution. The released EM and GP were collected from the release medium and measured using a UV-Vis absorption spectrometer (UV-1800, Shimadzu, Japan) after centrifugation at 13,000 rpm for 30 min. Drug release profiles in the absence of H_2_O_2_ were determined for comparison.

### Cell uptake

The human umbilical vein endothelial cell line (HUVEC) and mouse macrophage cell line (RAW264.7) were obtained from the Cell Bank of the Chinese Academy of Sciences. HUVECs and RAW264.7 cells were cultured separately in DMEM (Gibco) supplemented with 10% FBS (Gibco) and 1% penicillin-streptomycin (Invitrogen).

A transwell chamber was utilized to simulate the atherosclerotic microenvironment. HUVECs and RAW264.7 cells were cultured in the upper and lower chambers, respectively. Different NPs were labeled with Dil and added to the chambers after treating the cells with LPS (100 ng/mL) for 24 h. Fluorescence images of the cells incubated at 37 °C were captured 4 h later using a Leica microscope (Stellaris 5, Germany).

In another experiment, LPS-treated HUVECs and RAW264.7 cells were seeded into separate 12-well plates. Dil-labeled different materials (corresponding to lecithin concentrations of 100 µg/mL) were added for 4 h. The cells were also stained with 1 µM Lyso Tracker Green DND-99 for 1 h. Hoechst 33342 was used to stain the nuclei for 5 min, and fluorescence images were captured using a Nikon microscope (Ti-E + A1 MP, Japan).

For the immune escape assay, RAW264.7 cells were incubated with Dil-labeled different NPs (including LP NPs, MLP NPs, TK-LP NPs, and TK-MLP NPs) (corresponding to lecithin concentrations of 100 µg/mL) for 4 h. DAPI was used to stain the nuclei before capturing images under a Nikon microscope (Ti-E + A1 MP, Japan).

### In vitro inhibits HUVECS apoptosis of TK-MLP@(GP + EM) NPs

#### Analysis of apoptosis

After incubating HUVECs with 100 ng/mL LPS for 24 h, we further incubated the cells with GP + EM and TK-MLP@(GP + EM) NPs for an additional 24 h. The apoptosis of HUVECs was assessed using flow cytometry with an Annexin V-FITC/PI assay, following the manufacturer’s instructions (BD, CA, USA).

Subsequently, the cells were lysed, and the protein samples were subjected to SDS-PAGE gel separation. The separated proteins were then transferred onto a polyvinylidene fluoride (PVDF) membrane. The membranes were blocked with TBST containing 5% skim milk and incubated overnight at 4℃ with primary antibodies against Cleavedcaspase-3, Bcl-2, BAX, and β-actin. Following incubation, the membranes were washed and incubated with a secondary antibody at room temperature for 2 h. Finally, the membranes were washed and visualized using the BIO-RAD ChemiDoc XRS chemiluminescence system.

#### Detection of the function of HUVECs

HUVECs were seeded in 24-well plates. After 24 h of stimulation with LPS (100 ng/mL), GP + EM and TK-MLP@(GP + EM) NPs were added to the wells at concentrations of 15µM GP and 10 µM EM. The cells were then cultured for an additional 24 h. Following a blocking step of 30 min, primary antibodies against VE-cadherin were added and incubated overnight at 4℃. After washing with PBST three times, a fluorescence secondary antibody was added and incubated for 2 h. Prior to observation using a Nikon microscope (Ti-E + A1 MP, Japan), the nuclei were stained with DAPI. In addition, activated HUVECs were also subjected to immunofluorescence staining with anti-ICAM-1 antibody and CoraLite® Plus 488. Following the staining of nuclei with DAPI, the cells were observed using a Nikon microscope (Ti-E + A1 MP, Japan).

### In vitro improve lipid- removal of TK-MLP@(GP + EM) NPs

#### Inhibition of lipid influx

After a 24 h incubation with LPS (100 ng/mL), RAW264.7 cells were further treated with GP + EM and TK-MLP@(GP + EM) NPs, both at the same concentration of GP (15µM) and EM (10µM), for an additional 2 h. Subsequently, the cells were exposed to Dil-oxLDL (40 µg/mL) in the presence of fetal bovine serum and incubated for 4 h. Fluorescence images were then captured using confocal laser scanning microscopy (CLSM).

Furthermore, to assess the effects of different drugs, RAW264.7 cells were treated with LPS (500 ng/mL) for 2 h, followed by a 2 h incubation with the respective drugs. Subsequently, oxLDL (80 µg/mL) was added to the cell culture medium. After 48 h, the cells were stained with 0.3% ORO.

#### Promote cholesterol efflux

The impact of TK-MLP@(GP + EM) NPs on the levels of intracellular cholesterol excretion-related proteins ABCA1 and ABCG1 was assessed. RAW264.7 cells exposed to LPS stimulation, were treated separately with GP + EM and TK-MLP@(GP + EM) NPs for a duration of 24 h. Following treatment, the cells were collected and lysed. The resulting lysate was then subjected to centrifugation at 4℃ (12,000 rpm, 30 min), and the supernatant was collected for further Analysis. The expression levels of ABCA1 and ABCG1 were evaluated using western blotting, with the protein bands being semi-quantitatively analyzed using Image J software. Moreover, the expression of ABCA1 and ABCG1 was also examined using immunofluorescence and confocal laser scanning microscopy (CLSM) with the assistance of the Olympus FV1200 system.

### In vitro improve the mitochondrial function of TK-MLP@(GP + EM) NPs

#### Determination of mitochondrial morphology

Following the aforementioned treatment procedures, the cells were digested, harvested, and fixed with a solution containing 2.5% glutaraldehyde and osmium for a duration of 2 h. Subsequently, the cells underwent dehydration using a routine procedure involving a series of gradient ethanol solutions. Ultrathin slices were then prepared, and the structural changes of the mitochondria were examined using TEM.

#### JC-1 staining

Mitochondrial membrane potential was assessed using JC-1 dye staining obtained from Solarbio (China). This dye is capable of accumulating within the mitochondria in a manner dependent on mitochondrial membrane potential. It is then detected through a fluorescent emission shift from green (Ex/Em: 515/529 nm) to red (Ex/Em: 585/590 nm). The ratio of red fluorescence intensity to green fluorescence intensity serves as an indicator of mitochondrial depolarization, with a decreased ratio suggesting mitochondrial dysfunction. Both HUVECs and RAW264.7 cells were treated with JC-1 working solution (5 µg/mL) and incubated at 37 °C for 30 min. The cells were subsequently washed twice with JC-1 buffer, followed by observation using a Nikon microscope (Ti-E + A1 MP, Japan).

#### Assessment of mitochondrial ROS production

To visualize mitochondrial ROS through fluorescence microscopy, LPS was used to stimulate HUVECs and RAW264.7 cells, respectively. In order to avoid the destruction of TK-MLP NPs by a small amount of ROS induced by LPS, DMEM medium containing certain amino acid analogues was used to neutralize some ROS [31]. And we replaced the new medium before adding the nanomaterials. HUVECs and RAW264.7 cells were then treated with GP + EM and TK-MLP@(GP + EM) NPs for 24 h. Finally, the cells were incubated with a 10 µM MitoSOX™ Red probe at 37 °C in the dark for 30 min. Images of the cells were captured using a Nikon microscope (Ti-E + A1 MP, Japan).

### Pharmacokinetics and targeting capability

According to previous study [32], Ce6 can be used as an agent for fluorescence imaging in vivo due its good near-infrared fluorescence and low price. Therefore, we used Ce6 to label different nanomaterials to track the half-life, distribution, and aggregation of NPs in mice. In brief, blood samples from the eyelids of C57BL/6 mice were collected at various time points (0, 1, 2, 3, 4, 8, 12, and 24 h) following intravenous administration of different NPs treatments (5 mg/kg, Ce6). The serum samples were then analyzed using the IVIS spectroscopy system (Lumina XR). In addition, after being on a high-fat diet (HFD) for a duration of 10 weeks, atherosclerotic ApoE^−/−^ mice were injected intravenously with LP@Ce6 NPs, MLP@Ce6 NPs, and TK-MLP@Ce6 NPs (5 mg/kg, Ce6). After a 12-hour period, the aortas and other organs of the mice were measured using the IVIS spectroscopy (Lumina XR) technique.

### In vivo anti-atherosclerosis study

#### Treatment protocol

Male ApoE^−/−^ mice aged 6 weeks were subjected to a high-fat diet (HFD) for a duration of 4 weeks. The HFD comprised 40% fat, 40% carbohydrate, 20% protein, and 1.25% cholesterol (D12108C). Following this, the mice were treated with different interventions for a period of 8 weeks while remaining on the continuous HFD. The interventions included intravenous administration of saline, Mitoquinone (MitoQ) at a dosage of 5 mg/kg, a combination of GP + EM at a dosage of 5 mg/kg GP and 5 mg/kg EM, and TK-MLP@(GP + EM) NPs at a dosage of 5 mg/kg GP and 5 mg/kg EM.

#### Determination of atherosclerotic lesions and plaque histology

After completing the treatment, the mice were sacrificed, and the aortas were isolated. Pictures of the aortic arch and aortic root were captured using a mobile phone. Additionally, the entire aorta of ApoE^−/−^ mice was fixed with 4% paraformaldehyde until it became transparent. Subsequently, the longitudinally opened aortas were stained with 3% ORO to visualize lipid-rich plaques. The lesion area in the aorta, extending from the left common carotid artery to the iliac bifurcation, was evaluated. Serial cross-sections of the aortic root were prepared and stained with 3% ORO, as described in a previous study [33]. The quantification of the plaque area was performed using Image-Pro Plus 6.0 software.

Furthermore, the cross sections of the aortic root were subjected to H&E staining and Masson trichrome staining (Solarbio, China) to assess the area of necrotic cores, the thickness of fibrous caps, and collagen content. The levels of ROS in the plaques were determined by DHE staining (Solarbio).

#### Detection of the function of endothelial cells in atherosclerotic plaques

Immunofluorescent staining was performed on frozen cross-sections using primary antibodies targeting CD31, ICAM-1, and VE-cadherin to examine the presence of specific markers in the aortic root tissue. Following incubation with fluorescently labeled secondary antibodies, the sections were analyzed.

#### Detection of the lipid metabolism in atherosclerotic mice

Immunofluorescent staining was performed on frozen cross-sections of the aortic root using a primary antibody against F4/80. Subsequently, the sections were incubated with fluorescently labeled secondary antibodies for visualization. Additionally, immunohistochemical staining was carried out on paraffin-embedded cross-sections of the aortic root using primary antibodies against ABCA1 and ABCG1. The sections were then incubated with biotinylated goat anti-rabbit IgG, followed by fluorescence imaging using an inverted microscope (Olympus, IX-73).

### Safety evaluation

Following completion of the treatment, the mice were sacrificed, and major tissues, including the heart, liver, spleen, lung, and kidney, were promptly dissected. Subsequently, H&E staining was conducted to evaluate any pathological changes in these tissues. Moreover, blood samples from ApoE^−/−^ mice were collected in EDTA-spray-coated tubes post-treatment and were immediately subjected to analysis using an automated hematology analyzer (Sysmex TEK-VET3, Sysmex Co., China). Additionally, the levels of liver and renal function in the plasma of the mice were assessed using an automated analyzer platform (Roche Cobas C501, Roche Co., Switzerland), with the samples collected in heparin spray-coated tubes. These analyses aimed to ascertain any potential effects or alterations induced by the treatment on tissue morphology and the functioning of organs.

### Statistical analysis

Statistical Analysis was performed using GraphPad Prism version 8.3.0, employing one-way Analysis of variance (ANOVA) to calculate the significance of the data. All results were expressed as the mean value ± standard deviation and were derived from independent experiments.

## Results and discussion

### Fabrication and characterization of TK-MLP@(GP + EM) NPs

Endothelial cell dysfunction, characterized by apoptosis, is observed in the lesion regions of the arterial vasculature during the early stages of AS. This dysfunction plays a significant role in plaque regression and plaque instability [34]. In these areas, there is an enlargement of the space between injured endothelial cells, which leads to the accumulation of lipids beneath the endothelium. This lipid accumulation is taken up by macrophages, resulting in the formation of foam cells and promoting the progression of AS.

To address these issues, we investigated the potential of GP and EM as therapeutic agents. GP has been shown to inhibit apoptosis in HUVECs, while EM has been found to reduce lipid accumulation caused by macrophages [9, 15]. We employed flow cytometry and confocal microscopy to evaluate the effects of GP and EM on apoptosis in HUVECs and the uptake of Dil-oxLDL by macrophages. Interestingly, we found that GP exhibited a stronger ability to inhibit apoptosis in HUVECs, while EM showed a superior capacity to reduce the uptake of Dil-oxLDL by macrophages. Motivated by these findings, we combined GP and EM to enhance the therapeutic potential for AS. As expected, the combination of GP and EM resulted in a more pronounced inhibition of HUVECs apoptosis and macrophage uptake of Dil-oxLDL (Fig. S1). Furthermore, to mitigate the hepatorenal toxicity of the drugs, we opted for a lower concentration of GP and EM [35, 36].

**Fig. 1:**
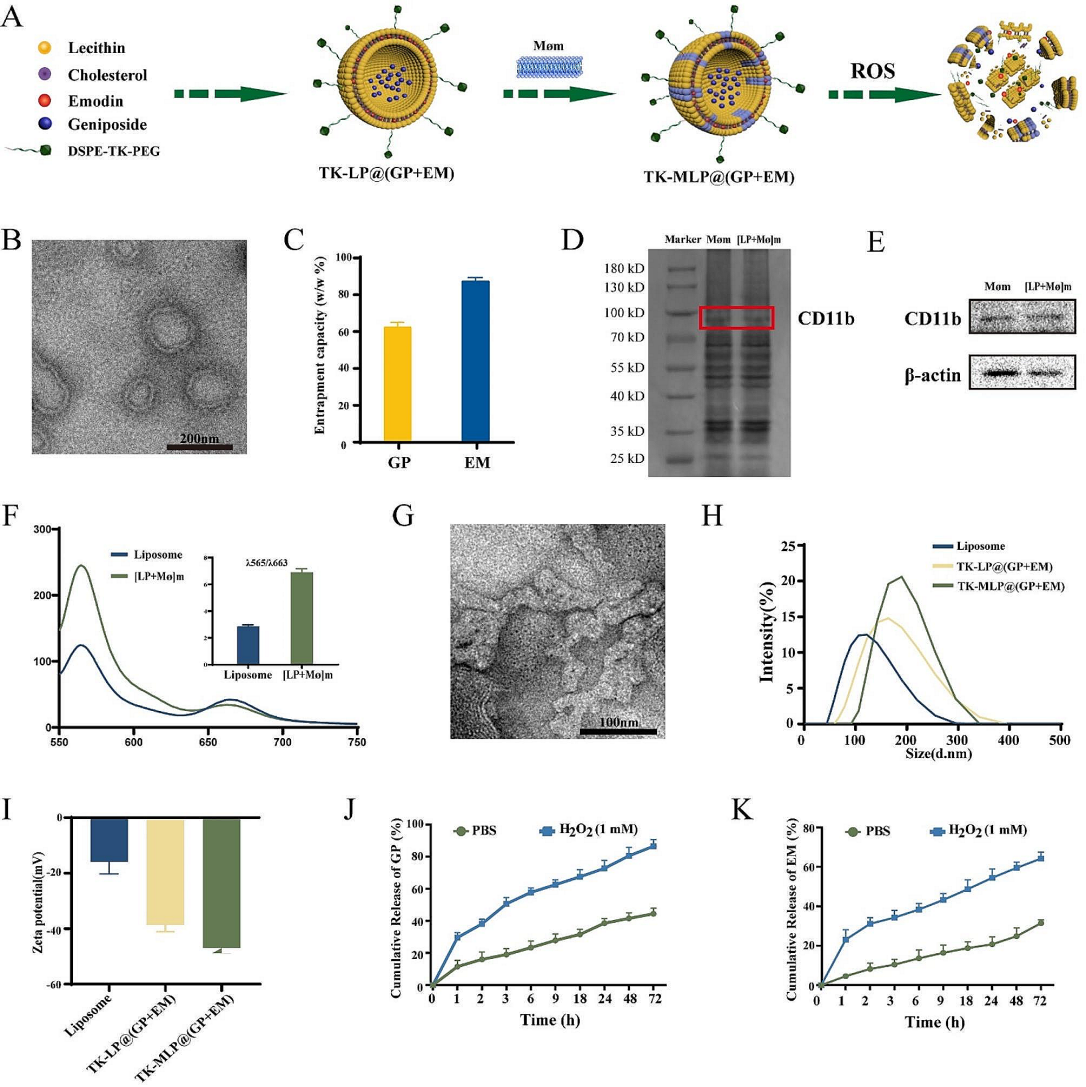
Characterization of TK-MLP@(GP + EM) NPs. (**A**) Schematic diagram of the synthetic process of TK-MLP@(GP + EM) NPs. (B) TEM image of TK-MLP@(GP + EM) NPs. (C) GP and EM entrapment efficiency of TK-MLP@(GP + EM) NPs. (D) SDS-PAGE analysis of retention protein bands of Møm and [LP + Mø]m NPs. (E) Western blot of Møm and [LP + Mø]m NPs for characteristic Møm marker CD11b. (F) The infusion efficiency investigation of Liposome NPs and [LP + Mø]m. The fluorescence recovery of DiI (565 nm) represented the fusion of LP NPs and [LP + Mø]m. (G) TEM image of TK-MLP@(GP + EM) NPs after being treated with 1 mM H_2_O_2_ solution for 4 h. (H&I) Particle size and zeta potential of TK-MLP@(GP + EM) NPs analyzed by DLS. (J&K) Release profile investigation of GP and EM in PBS and H_2_O_2._

The liposomes were prepared using the thin film hydration method, as outlined in Fig. 1A. GP and EM were loaded into the LP NPs and modified with DSPE-TK-PEG_2000_. The fusion of Møm hybridization forms TK-MLP@(GP + EM) NPs. TEM images confirmed the spherical morphology of TK-MLP@(GP + EM) NPs, which were surrounded by a thin film coating resulting from the macrophage cell membrane hybridization (Fig. 1B). To accommodate the difference in solubility between GP and EM, LP NPs were loaded with a feed ratio of 1:20 (drug: lecithin), allowing EM to be incorporated into the phospholipid layer and GP into the core. The encapsulation efficiency percentages for GP and EM were determined to be 87.4% and 62.5%, respectively (Fig. 1C). In addition, the SDS-PAGE assay confirmed the preservation of membrane proteins in both Møm and TK-MLP@(GP + EM) NPs (Fig. 1D). Western blotting analysis further confirmed the existent of specific bands of CD11b in TK-MLP@(GP + EM) NPs, the corresponded protein marker for Møm (Fig. 1E). Furthermore, we adopted Förster resonance energy transfer (FRET) experiment to further verify the fusion of Møm and Liposome. Fig. 1F showed that compared with LP NPs alone, with the fusion of Liposome membrane and Møm, the trend of fluorescence intensity change at 565 nm is opposite to that at 663 nm as the fusion of the Liposome membrane and Møm leads to the separation of the DiI/DiD energy resonance transfer pair of the fluorophore, thus restoring the fluorescence signal of DiI (565 nm).

In the AS pathological environment, an H_2_O_2_ concentration of 1 mM is considered indicative of oxidative stress [28]. Therefore, the morphology changes of TK-MLP@(GP + EM) NPs were observed by TEM after incubation in a 1 mM H_2_O_2_ solution for 4 h (Fig. 1G). The bilayer and cavity structure vanished, the structure of NPs broke up into fragments, indicating the strong response of the nanoparticles to ROS. This suggests that ROS can serve as an intelligent switch to trigger the disintegration of TK-MLP@(GP + EM) NPs. Additionally, the average diameter of TK-MLP@(GP + EM) NPs, as measured by DLS, was approximately 184.6 nm (Fig. 1H), with a zeta potential of -46.93 mV (Fig. 1I). The cumulative drug release rates of GP and EM from TK-MLP@(GP + EM) NPs in PBS over 72 h were found to be 44.4% and 31.4%, respectively. In contrast, TK-MLP@(GP + EM) NPs disintegrated and dissociated in the presence of 1 mM H_2_O_2_ due to the robust ROS response mediated by TK. As a result, the cumulative drug release rates of GP and EM reached 86.5% and 64.2% after 72 h, respectively (Fig. 1J&K). Overall, these results demonstrate that TK-MLP@(GP + EM) NPs have the ability to dissolve and efficiently release drugs in the high ROS microenvironment at the AS site rapidly and efficiently.

### Cellular uptake of TK-MLP@(GP + EM) NPs

Macrophages play a crucial role in the innate immune system. It was observed in Fig. S2 that the red fluorescence signal in macrophages treated with MLP NPs was significantly lower than that in the LP NPs group and TK-LP NPs group. This phenomenon was also visible in the red fluorescence signal of the TK-MLP NPs group, indicating that Møm possesses the ability to prevent macrophages from phagocytosing TK-MLPs. This can be attributed to the camouflage ability of Møm, which has been reported in previous study [37]. Additionally, we found that LPS-treated HUVECs exhibited a strong fluorescence signal after incubating with TK-MLP@Dil NPs, compared to intact HUVECs (Fig. S3). This result suggests that TK-MLP@(GP + EM) NPs can target injured endothelial cells, which is attributed to the interaction between the high ICAM-1 expression of activated endothelial cells and the CD11b surface molecule present on Møm [38].

**Fig. 2:**
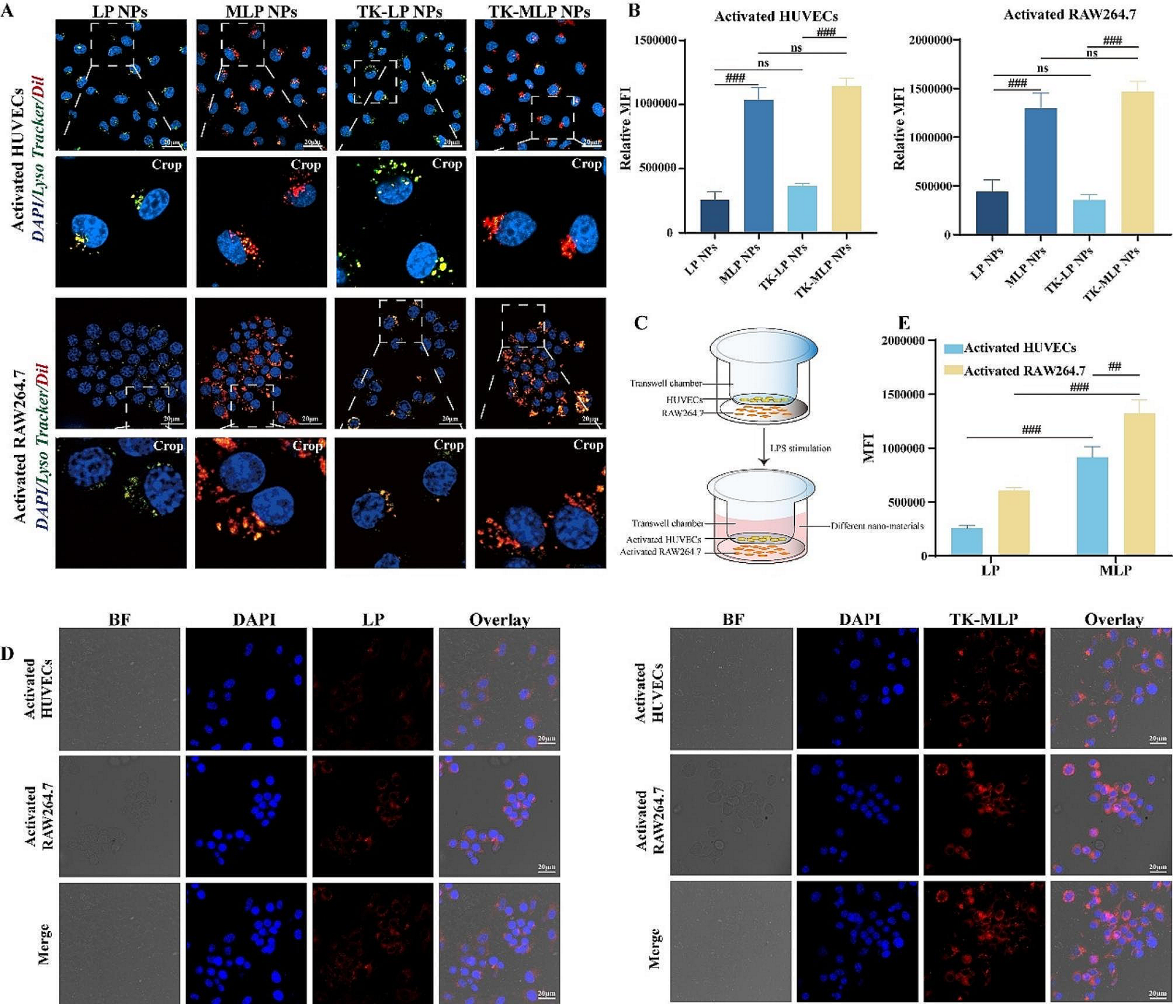
Cellular uptake of TK-MLP NPs. (A&B) Representative fluorescence images and quantitation of activated HUVECs and RAW264.7 cells 4 h after incubation with LP, MLP, TK-LP NPs, and TK-MLP NPs (red). Green represents the Lyso Tracker. Scale bar = 20 μm. (C)The schematic diagram showed that HUVECs were co-cultured with RAW264.7 cells in a transwell system for simulating plaque in vitro. (D&E) Phagocytosis and quantitation of LP NPsand TK-MLP NPs in activated HUVECs and RAW264.7 cells in transwell. BF indicates bright field. Scale bar = 20 μm. *n* = 3, ^##^*P* < 0.01, ^###^*P* < 0.001.

As the “digestive organ” within cells, lysosomes play a role in the degradation and elimination of drugs, which can reduce their therapeutic effects [39]. Upon studying the interaction between TK-MLP NPs and lysosomes in activated HUVECs and macrophages, the uptake of TK-MLP NPs group by activated macrophages and HUVECs was highly efficient, surpassing that of the LP NPs group and TK-LP NPs group, while the green fluorescence of the lysosomal probe was observed. We discovered that the weak red fluorescence in the LP NPs and TK-LP NPs group was notably, this observation was consistent in both LPS-treated HUVECs and macrophages. Additionally, the yellow fluorescence signal (represents red fluorescence exhibited co-localization with the lysosomes) of the TK-LP NPs group was significantly stronger than that of the TK-MLP NPs group, suggesting that the hybridization of Møm facilitates the bypassing of lysosomes and internalization of NPs into activated endothelial cells and macrophages within the AS environment, provides a foundation for the precise and targeted therapeutic application of drugs (Fig. 2A&B).

To directly assess the cellular uptake of NPs within atherosclerotic plaques, a transwell system was employed, wherein LPS-treated HUVECs were incubated in the upper chamber, and activated macrophages were cultured in the lower chamber (Fig. 2C) [40]. The results displayed in Fig. 2D&E demonstrated that the red fluorescence within both activated macrophages and HUVECs was notably stronger in the TK-MLP group than in the LP group. These findings indicate that TK-MLP NPs can be effectively internalized by HUVECs and macrophages within the pathological environment characterized by heightened levels of ROS, attributed to the targeting and “homing effect” of Møm [25]. Moreover, these results establish a foundation for investigating the molecular mechanisms underlying the potential therapeutic applications of nanomaterials in atherosclerosis treatment.

### TK-MLP@(GP + EM) NPs can inhibit the apoptosis of HUVECs

**Fig. 3:**
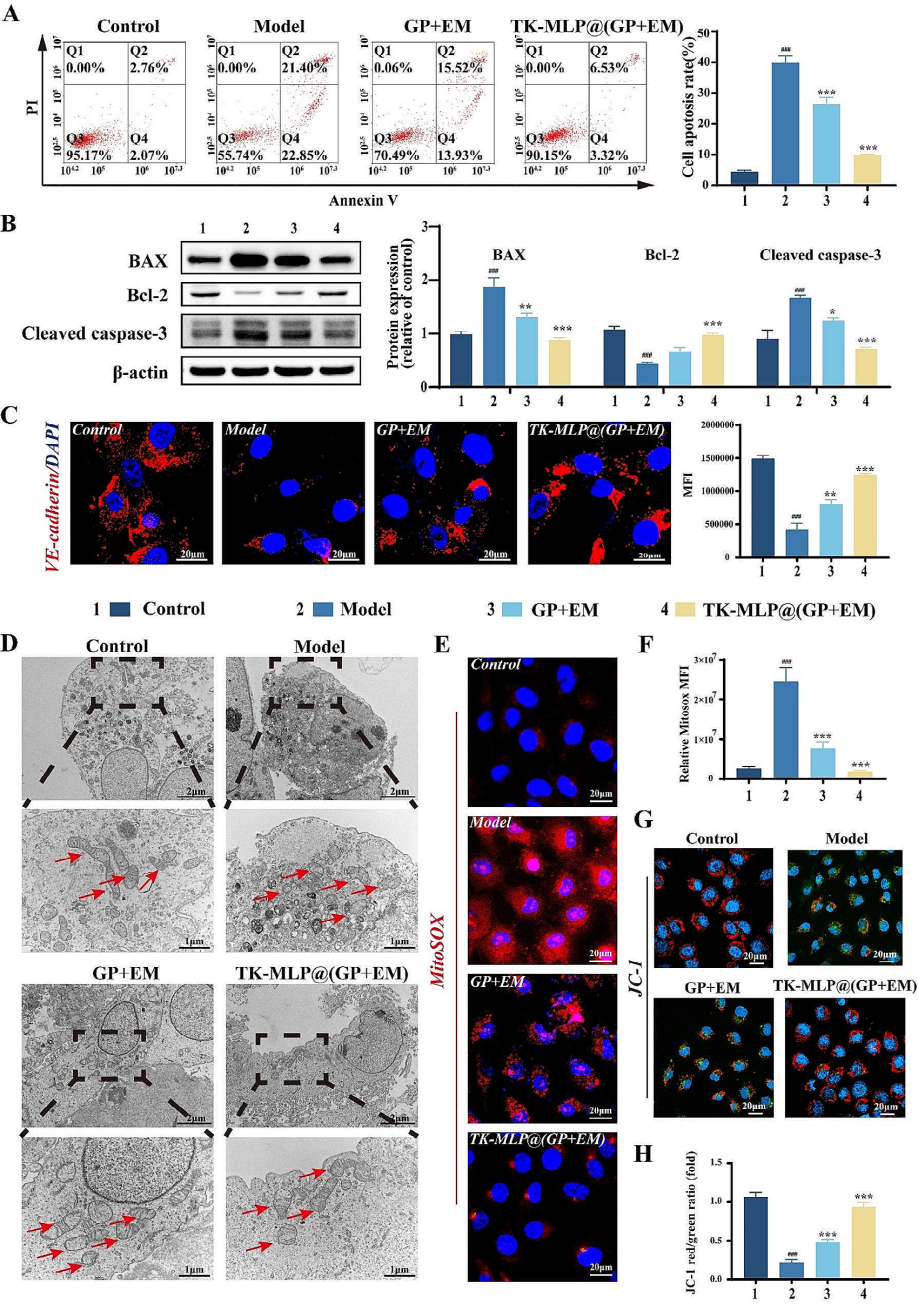
TK-MLP@(GP + EM) NPs can inhibit the apoptosis of endothelial cells. (A) Apoptosis detection after various treatments by flow cytometry. (B) Western blotting assay of BAX, Bcl-2, and Cleaved caspase-3 levels in HUVECs with different treatments. (C) Confocal laser scanning microscopy images of VE-cadherin in HUVECs with different treatments. (D) The ultrastructure of mitochondria in HUVECs was observed using a transmission electron microscope (TEM). The arrows point to different states of mitochondrial structure. (E&F) Mitochondrial superoxide levels by MitoSOX Red fluorescent staining with MFI quantification. (G&H) Mitochondrial membrane potential by JC-1 staining with calculation of the ratio of red MFI (aggregated JC-1) to green MFI (monomer JC-1). 1: Control;2: Model; 3: GP + EM; 4: TK-MLP@(GP + EM). Scale bar = 20 μm. *n* = 3, ^###^*P* < 0.001 vs. the Control. ** *P* < 0.01, *** *P* < 0.001 vs. the Model.

Previous study has demonstrated that LPS can induce apoptosis in endothelial cells [41]. The apoptosis of endothelial cells leads to dysfunction, damage to the cell barrier, secretion of adhesion factors, and recruitment of monocytes to form macrophages, thereby contributing to the development and progression of atherosclerosis [42]. Thus, in this study, we utilized LPS-stimulated HUVECs as a model to investigate the effects of TK-MLP@(GP + EM) NPs on apoptosis. Flow cytometry analysis revealed that both early and late apoptosis of HUVECs in the Model group (∼ 21.4% and ∼ 22.85%, respectively). Compared with the Model group, TK-MLP@(GP + EM) NPs resulted in a significant ∼ 19.5% decrease in the early apoptosis rate and ∼ 14.9% decrease in the late apoptosis rate, respectively (Fig. 3A). Furthermore, western blot analysis demonstrated that the treatment with TK-MLP@(GP + EM) NPs up-regulated the expression of the antiapoptotic protein Bcl-2 and down-regulated the expression of the proapoptotic protein BAX. Additionally, Fig. 3B showed a 1.8-fold increase in Cleaved caspase-3 levels, a mediator of apoptosis, in HUVECs following LPS stimulation, consistent with previous reports [41]. However, treatment with TK-MLP@(GP + EM) NPs inhibited the expression of Cleaved cjaspase-3 (Fig. 3B). Collectively, these results further support the notion that TK-MLP@(GP + EM) NPs inhibit apoptosis in HUVECs.

LPS can change cell morphology and induce overexpression of intercellular cell adhesion molecule 1 (ICAM-1) in HUVECs by promoting apoptosis [43]. Phalloidin immunofluorescent staining revealed that LPS altered the morphology of HUVECs from cobblestone-like shapes to spindle shapes, which was attenuated by treatment with TK-MLP@(GP + EM) NPs. Moreover, TK-MLP@(GP + EM) NPs down-regulated the expression of ICAM-1 (Fig. S4). VE-cadherin, an endothelium-specific adhesion molecule, plays a crucial role in maintaining the integrity of endothelial cells and preventing AS [44]. We observed a significant 70% reduction in the expression of VE-cadherin (red fluorescence) in activated HUVECs. However, treatment with TK-MLP@(GP + EM) NPs resulted in a substantial 67% increase in the expression of VE-cadherin, compared to the Model group (Fig. 3C). These findings indicate that TK-MLP@(GP + EM) NPs are capable of reversing LPS-induced apoptosis and repairing the damage to HUVECs.

Endothelial cell apoptosis is primarily induced by excessive production of ROS resulting from mitochondrial damage [45]. Therefore, we utilized TEM to examine the ultrastructure of HUVECs mitochondria. In the Model group, we observed mitochondrial enlargement, swelling, hypodense matrix, and vacuolar degeneration of mitochondrial cristae. In contrast, the TK-MLP@(GP + EM) NPs group exhibited clear and well-preserved ultrastructure of endothelial mitochondria, characterized by dense mitochondrial cristae and a matrix with normal density (Fig. 3D). Subsequently, we investigated the impact of TK-MLP@(GP + EM) NPs on mitochondrial ROS in activated HUVECs by using fluorescent dyes MitoSOX. As demonstrated in Fig. 3E&F, the red fluorescence signal in activated HUVECs was significantly enhanced approximately 9.2 times compared to the Control group. However, the accumulation of mitochondrial ROS induced by LPS was mitigated by treatment with TK-MLP@(GP + EM) NPs, as evidenced by a remarkable 92% reduction in red fluorescence intensity. We attribute this reduction to the fact that the abundance of mitochondrial ROS triggered the breakdown of TK, consequently leading to the rapid release of the drugs. Additionally, mitochondrial dysfunction is characterized by a decrease in membrane potential [46]. Therefore, we employed JC-1 staining to evaluate the transmembrane potential (Δψm) of HUVECs. A higher red/green ratio of JC-1 fluorescence indicates a lower degree of mitochondrial dysfunction. LPS stimulation significantly lowered the Δψm of HUVECs. However, this impairment was significantly ameliorated after treatment with TK-MLP@(GP + EM) NPs, as demonstrated by a remarkable increase in the red/green ratio (Fig. 3G&H). These results suggest that TK-MLP@(GP + EM) NPs possess potent capability in maintaining the normal membrane potential of mitochondria. Hence, TK-MLP@(GP + EM) NPs exhibit the potential to inhibit HUVECs apoptosis resulting from mitochondrial damage and aid in the recovery of HUVECs functionality.

### TK-MLP@(GP + EM) NPs can inhibit lipid accumulation of macrophages

**Fig. 4:**
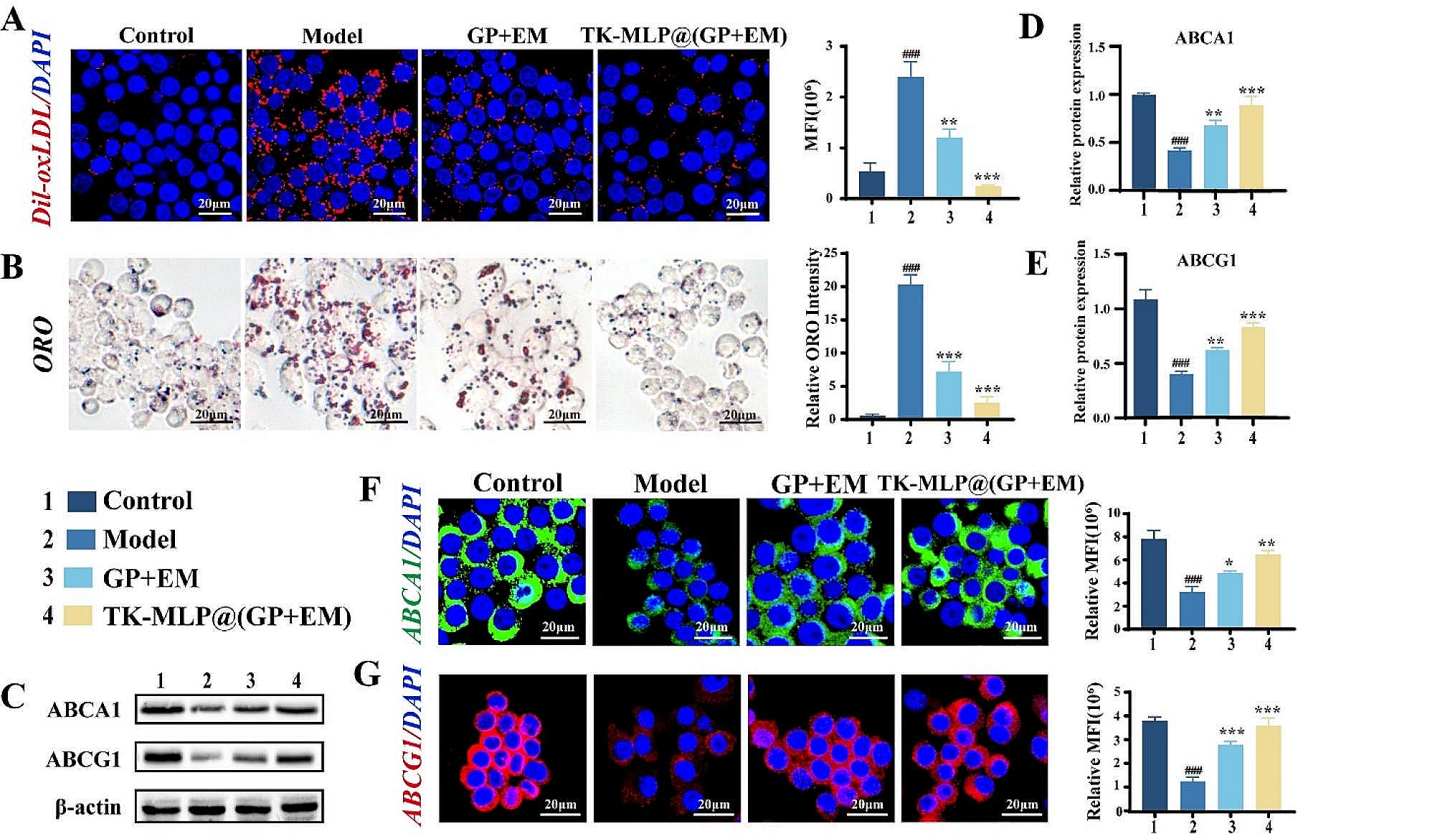
TK-MLP@(GP + EM) NPs inhibits lipid accumulation of macrophages. (A) Confocal fluorescence images and semi-quantitative analysis of DiI-oxLDL internalization in RAW264.7 cells. (B) Optical microscopy images and semi-quantitative analysis of ORO staining. (C-E) Western blot analysis of ABCA1 and ABCG1 on activated macrophages with different treatment. (F&G) Confocal fluorescence images of ABCA1 and ABCG1 on activated macrophages with different treatment. 1: Control;2: Model; 3: GP + EM; 4: TK-MLP@(GP + EM). Scale bar = 20 μm. *n* = 3, ^###^*P* < 0.001 vs. the Control. *P < 0.05, ** *P* < 0.01, *** *P* < 0.001 vs. the Model

The apoptosis of endothelial cells can have detrimental effects on the development of AS by causing further lipid accumulation and accelerating the progression of the lesion [47]. In this study, we also examined the impact of TK-MLP@(GP + EM) NPs on lipid deposition. We investigated the reduction of lipid uptake by activated macrophages by observing the change in fluorescence signal using Dil-oxLDL. The Model group exhibited a strong red fluorescence signal, indicating significant uptake of Dil-oxLDL by activated macrophages. However, in the TK-MLP@(GP + EM) NPs group, we observed a remarkable decrease in red fluorescence compared to the Model group, suggesting a better reduction in Dil-oxLDL uptake by activated macrophages (Fig. 4A). Furthermore, we assessed intracellular lipid droplets in activated macrophages using ORO staining. The results demonstrated a reduction in intracellular lipid droplets with TK-MLP@(GP + EM) NPs treatment, which can be attributed to the inhibition of ox-LDL internalization (Fig. 4B).

Considering that ABCA1 and ABCG1 play a crucial role in promoting cholesterol excretion during the development of atherosclerosis [48], we examined the effects of TK-MLP@(GP + EM) NPs on the expression of these transporters in activated macrophages. Compared to the Control group, LPS led to down-regulation of ABCA1 and ABCG1 levels in macrophages, which is consistent with our previous study [32]. However, treatment with TK-MLP@(GP + EM) NPs resulted in up-regulation of both ABCA1 and ABCG1 protein expression levels (Fig. 4C-E). Moreover, confocal microscopy images also demonstrated a significant increase in the expression of ABCA1 and ABCG1 with TK-MLP@(GP + EM) NPs treatment (Fig. 4F&G). These results indicate that TK-MLP@(GP + EM) NPs can effectively reduce lipid deposition by modulating lipid internalization and efflux. Previous study confirmed that re-polarizing macrophages from M1 to M2 type is beneficial for the treatment of AS [25], we subsequently evaluated the capability of TK-MLP@(GP + EM) NPs for reprogramming macrophages. In Fig. S5A&B, flow cytometry assay showed the signal decrease of CD80 while increase for CD206 in activated macrophages in the GP + EM group, because both GP and EM can induce M1-to-M2 repolarization of macrophages [20, 49]. What is more, the best therapeutic effect of TK-MLP@(GP + EM) NPs can attribute to the function of TK-MLP NPs on consuming high ROS levels in the Model group [50]. Therefore, these results indicate that TK-MLP@(GP + EM) NPs could efficiently reduce lipid deposition by inducing M1-to-M2 re-polarization of macrophages.

It has been established that the transformation of M1 macrophage is accompanied by mitochondrial dysfunction [51]. To assess mitochondrial integrity and condition among different groups, we observed it through TEM. As shown in Fig. S5C, mitochondria in the Control group appeared intact with clear mitochondrial crista, while the Model group displayed more swollen and damaged mitochondria. However, treatment with TK-MLP@(GP + EM) NPs resulted in the recovery of a clear and intact mitochondrial structure. Furthermore, we investigated the impact of TK-MLP@(GP + EM) NPs on mitochondrial ROS and Δψm in activated macrophages. As depicted in Fig. S5D&E, treatment with TK-MLP@(GP + EM) NPs attenuated the accumulation of mito-ROS caused by LPS by approximately 90% and restored mitochondrial Δψm. These results suggest that TK-MLP@(GP + EM) NPs can promote the repolarization of macrophages from M1 to M2 type, which is achieved by restoring mitochondrial function.

Overall, our findings provide further evidence that TK-MLP@(GP + EM) NPs possess the ability to reverse M1 macrophage polarization by preventing mitochondrial damage, eventually inhibit lipid accumulation and intervene atherosclerosis development.

### Pharmacokinetics and targeting capability of TK-MLP@(GP + EM) NPs

**Fig. 5:**
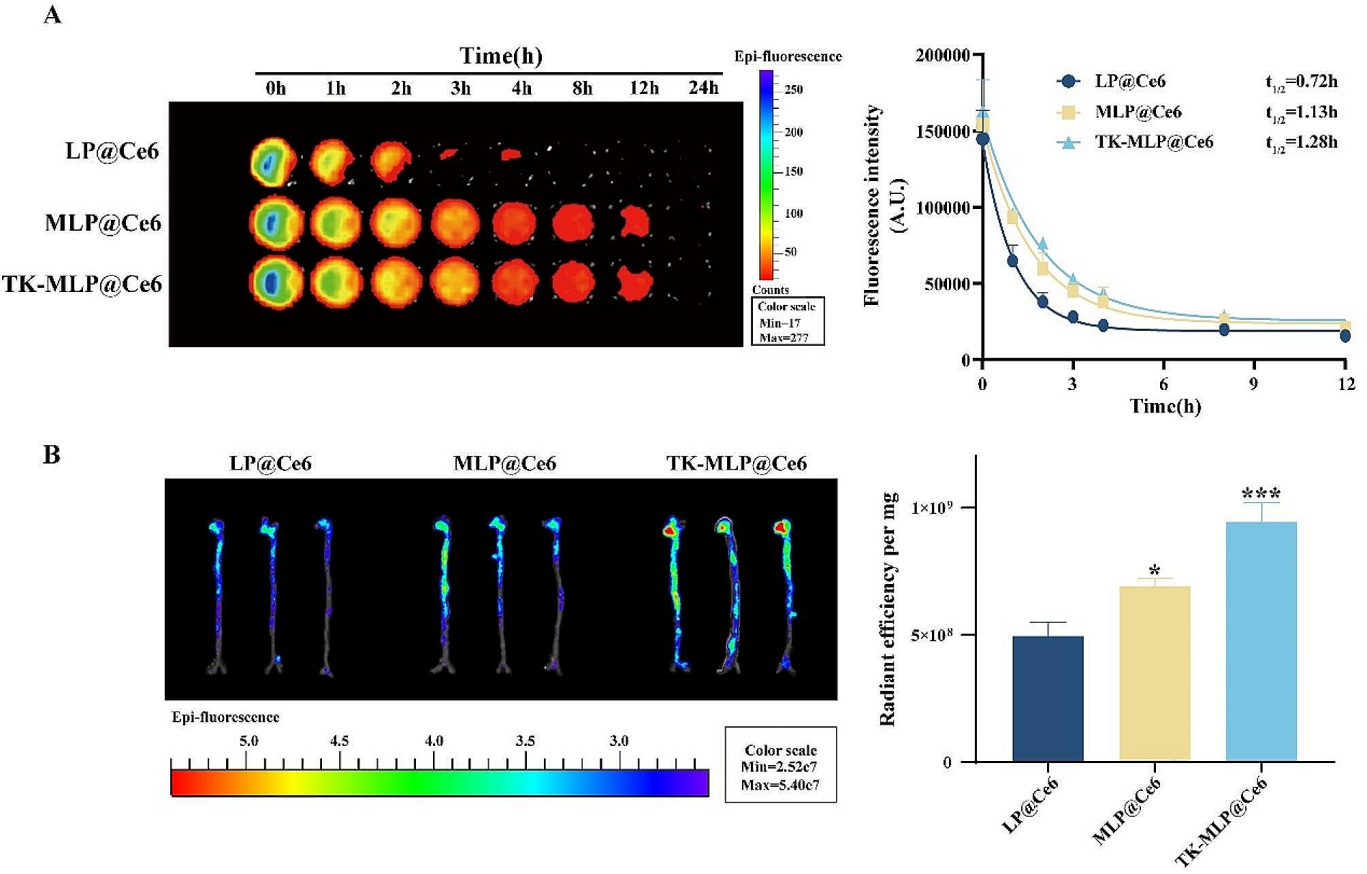
Pharmacokinetics and targeting capability of TK-MLP NPs. (A) Representative photographs of blood samples collected from C57BL/6 mice after administration of different nano-materials at various time points. Pharmacokinetic curves of different nano-materials. *n* = 3. (B) Fluorescence photos and semi-quantitative show the fluorescent signals of Ce6 in aortas from ApoE^−/−^ mice. ApoE^−/−^ mice fed with HFD for 2 months were intravenously injected with different NPs. After administration of 12 h, the aortas of ApoE^−/−^ mice were isolated for detection. *n* = 3. *P < 0.05, ***P < 0.001 vs. the LP@Ce6.

To assess the prolonged circulation time of TK-MLP NPs, we conducted pharmacokinetic studies in male wild-type C57BL/6 mice. Ce6-labeled NPs were intravenously injected, and the residual content of nanoparticles was measured by analyzing the fluorescence intensity of Ce6 in blood samples collected at various time intervals. Compared to LP@Ce6 NPs with a circulation half-life (t_1/2_) of approximately 0.72 h, fluorescence imaging revealed that both MLP@Ce6 NPs and TK-MLP@Ce6 NPs exhibited extended blood circulation (t_1/2_ ≈ 1.13 h for MLP@Ce6 NPs; t_1/2_ ≈ 1.28 h for TK-MLP@Ce6 NPs), which is consistent with previous findings [23] (Fig. 5A). These results highlight the ability of membrane coating to prolong the circulation time of NPs, which is beneficial for enhancing drug accumulation in atherosclerotic plaques.

To assess the targeting capability of TK-MLP@(GP + EM) NPs, ApoE^−/−^ atherosclerotic mice were injected intravenously with TK-MLP@Ce6 NPs to track its accumulation in the mice. As depicted in Fig. 5B, the fluorescence intensity of MLP@Ce6 NPs at the aortic site was observed to increase by approximately 1.40 times compared to LP@Ce6 NPs. This can be attributed to the high affinity between ICAM-1 on damaged endothelial cells and CD11b receptors on the macrophage cell membranes [52]. Furthermore, the signal intensity of the TK-MLP@Ce6 NPs treatment group increased by approximately 1.91 times. This enhanced targeting ability can attribute to the presence of abnormal ROS in atherosclerotic plaques, which effectively break the TK bonds of nanomaterials to trigger the release of Ce6. These results demonstrate that the Møm camouflaging and ROS responsiveness of TK significantly enhance the targeting and release abilities of TK-MLP@(GP + EM) NPs in ApoE^−/−^ mice.

NPs are known to accumulate in the liver due to the first-pass effect, which involves the uptake of NPs by macrophages [53]. In our study, we observed that the fluorescent signal of the liver was higher in the LP@Ce6 NPs group compared to the MLP@Ce6 NPs and TK-MLP@Ce6 NPs groups (Fig. S6). This indicates that Møm coating is beneficial in preventing liver elimination and increasing the accumulation of TK-MLP@(GP + EM) NPs in plaques. Moreover, a strong fluorescence signal was observed in the kidneys of mice for all NPs, including TK-MLP@(GP + EM) NPs, due to the metabolism and excretion of NPs through the kidneys. Overall, these findings suggest that TK-MLP@(GP + EM) NPs have the ability to chronically localize and accumulate in atherosclerotic plaques.

### The efficacy of TK-MLP@(GP + EM) NPs in ApoE^-/-^ mice fed HFD

**Fig. 6:**
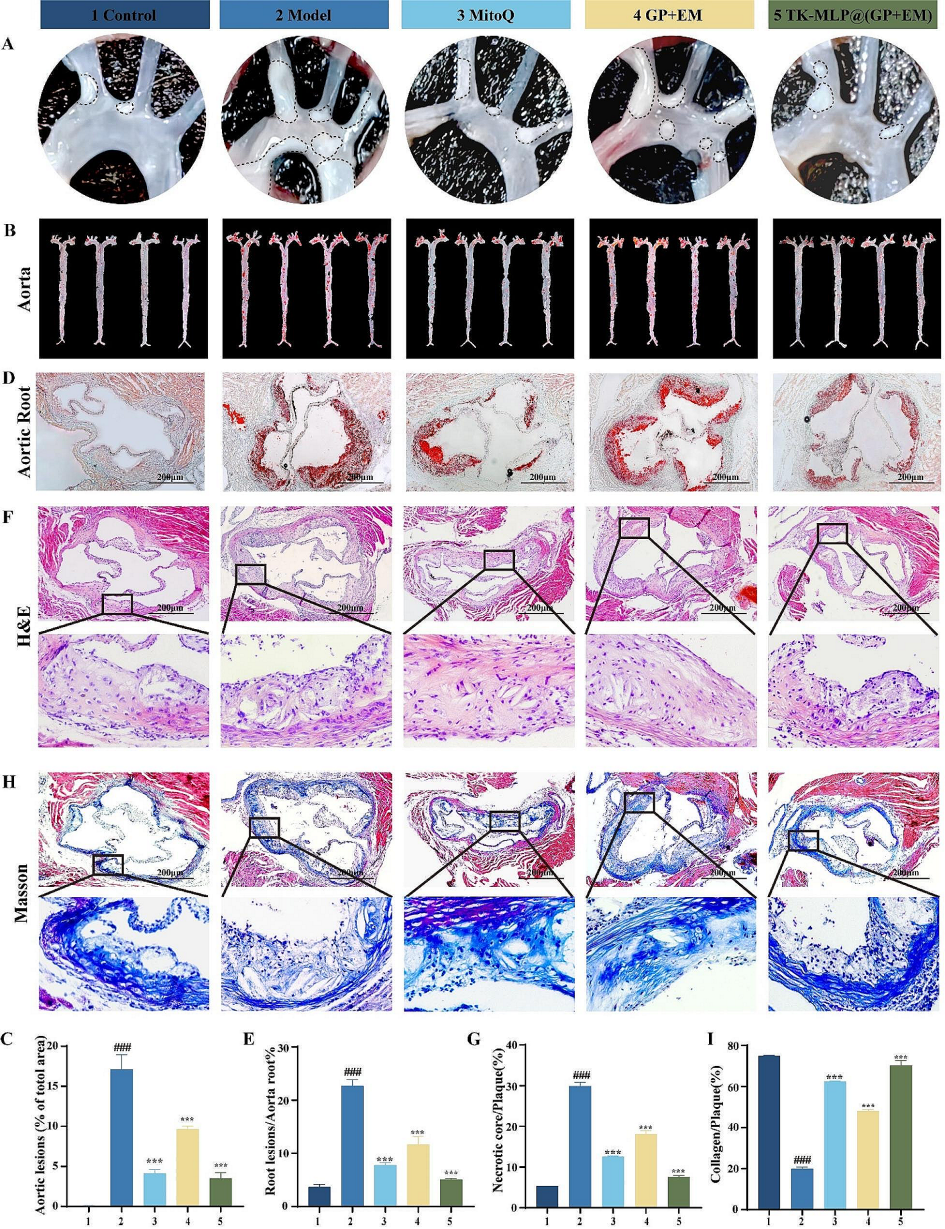
The efficacy of TK-MLP@(GP + EM) NPs in ApoE^−/−^ mice fed HFD. (A) Photographs of the aortic arch. (B&C) The representative images of en face ORO-stained aortas and semi-quantitative analysis. *n* = 4. (D&E) ORO-stained frozen sections and semi-quantitative analysis of the aortic roots. (F-I) Representative immunohistochemistry staining photographs and semi-quantitative analysis with H&E and Masson trichrome. Scale bar = 200 μm. *n* = 3, ^###^*P* < 0.001 vs. the Control. *** *P* < 0.001 vs. the Model.

Based on the promising results obtained thus far, we further investigated the therapeutic effect of TK-MLP@(GP + EM) NPs on atherosclerotic plaques in vivo. Following treatment, we isolated the aorta and observed a significant reduction in plaque size in the aortic arch region (area circled by the black dotted line) (Fig. 6A). Additionally, ORO staining revealed that the area of lipid deposition in the plaques was approximately 1.06% and 17.15% in the Control and Model groups, respectively, confirming successful construction of the atherosclerosis model. Notably, when compared to the Model group, the plaque area was significantly reduced in ApoE^−/−^ mice treated with TK-MLP@(GP + EM) NPs. It is worth mentioning that in our animal experiments, we used MitoQ, a mitochondria-targeting antioxidant, as a positive group. The MitoQ group exhibited a significant reduction in plaque area (approximately 4.10%), indicating the potential of inhibiting mitochondrial oxidative stress for effective atherosclerosis treatment. Excitingly, TK-MLP@(GP + EM) NPs demonstrated a stronger inhibitory effect on aortic plaque formation compared to the free GP + EM group (plaque area approximately 9.64%). The plaque area with TK-MLP@(GP + EM) NPs treatment was approximately 3.50%, which was similar to the effect observed in the MitoQ group. This suggests that one of the mechanisms by which TK-MLP@(GP + EM) NPs reduce atherosclerotic plaques might be through the inhibition of mitochondrial oxidative stress (Fig. 6B&C). Furthermore, we investigated the impact of TK-MLP@(GP + EM) NPs on plaque formation in the high-occurrence region of the aortic roots. ORO staining of frozen sections revealed significant lipid deposition in the plaques of the Model group (approximately 22.68%), whereas TK-MLP@(GP + EM) NPs exhibited a significant anti-lipid deposition effect in all segments of the aortas (approximately 5.07%) (Fig. 6D&E).

Increased levels of ROS play a crucial role in the growth of the necrotic core, which is a key characteristic of atherosclerosis. In this study, the presence of large necrotic cores in the aorta root of the Model group was confirmed through H&E staining (Fig. 6F&G). However, treatment with TK-MLP@(GP + EM) NPs resulted in a significant reduction in necrotic cores by approximately 22.3%. Given that the extent of necrotic cores is positively associated with plaque vulnerability, Masson’s trichrome assay was performed to evaluate the stability of the plaques in ApoE^−/−^ mice. Among the various groups investigated, TK-MLP@(GP + EM) NPs exhibited the highest collagen content and fibrous cap thickness surrounding the plaques (Fig. 6H&I).

Taken together, these findings demonstrate the remarkable therapeutic effects of TK-MLP@(GP + EM) NPs on atherosclerosis.

### TK-MLP@(GP + EM) NPs can restore endothelial function in ApoE^-/-^ atherosclerosis mice

**Fig. 7:**
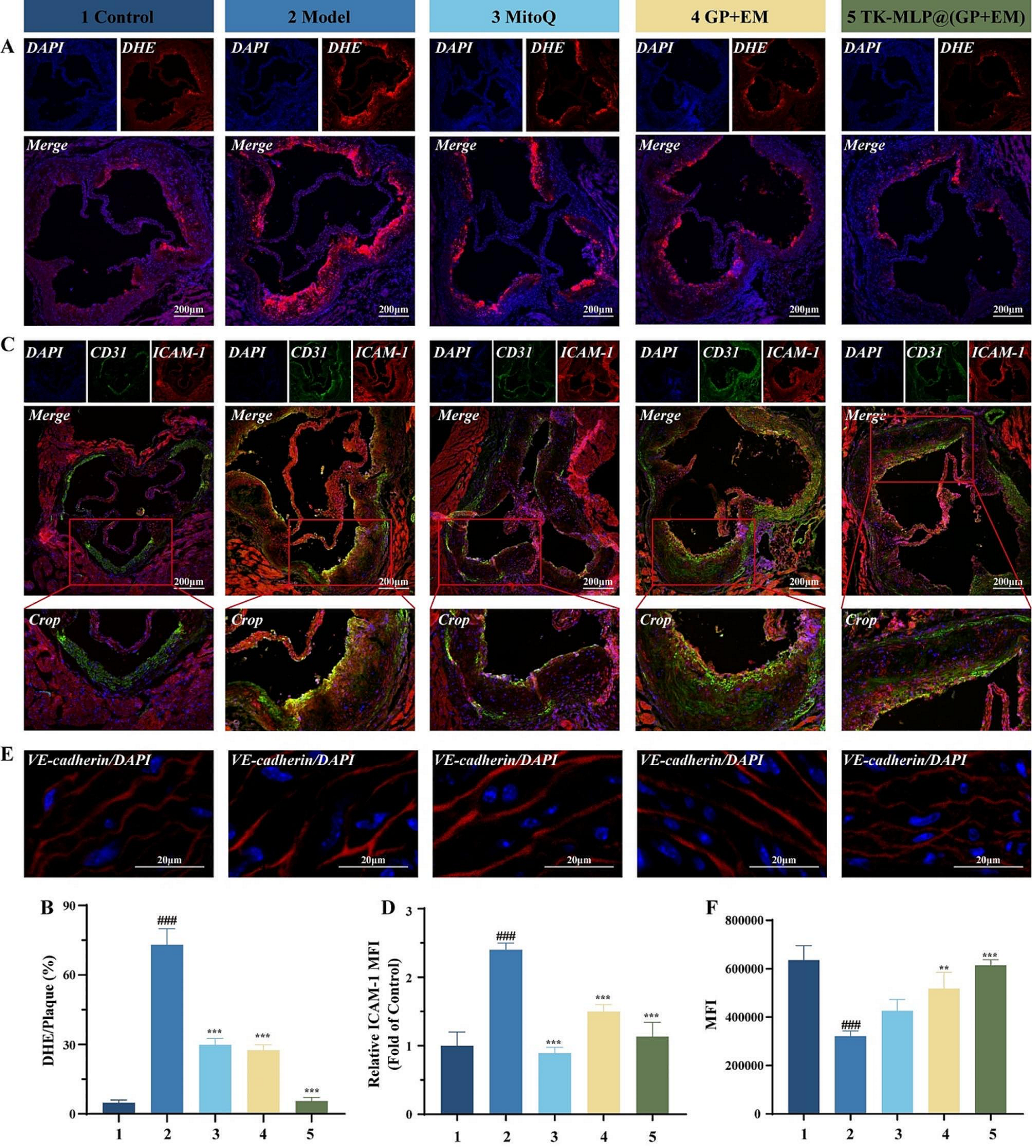
TK-MLP@(GP + EM) NPs can restore endothelial function in ApoE^−/−^ atherosclerosis mice. (A&B) Aortic root cross-sections were subjected to dihydroethidium staining for determination of the redox state with quantitative analysis of DHE MFI. (C&D) Aortic root cross-sections were conducted co-immunofluorescent staining with anti-CD31 (green) and ICAM-1 (red). (E&F) Immunofluorescence staining images of VE-cadherin in aortas from each group. Scale bar = 200 μm. *n* = 3, ^###^*P* < 0.001 vs. the Control. ** *P* < 0.01, *** *P* < 0.001 vs. the Model.

Previous studies have highlighted the presence of elevated levels of ROS in atherosclerotic plaques in ApoE^−/−^ mice [54]. In this study, DHE staining was performed on the freshly frozen aortic roots of ApoE^−/−^ mice to assess the ROS content within the plaques (Fig. 7A&B). Notably, the Model group exhibited a strong red fluorescent signal, indicative of high ROS levels in the aortic root plaques compared to the Control group. In alignment with previous finding [54], treatment with MitoQ resulted in a ∼ 43% reduction in ROS levels. Similarly, the GP + EM group and the TK-MLP@(GP + EM) NPs group also demonstrated varying degrees of ROS reduction, consistent with the in vitro ROS level detection results shown in Figs. 3E and 4G. Importantly, it is worth noting that the TK-MLP@(GP + EM) NPs group exhibited superior ROS elimination capability and the weakest fluorescence intensity, exceedingly even that of the MitoQ group. These results further support the notion that TK-MLP@(GP + EM) NPs can be thought of as similar anti-mitochondrial oxidants. The abundant ROS levels within the plaques can trigger the breakdown of DSPE-TK-PEG_2000_, facilitating the release of the encapsulated drugs, which subsequently accumulate within the plaques. Overall, these findings provide evidence of the potent ability of TK-MLP@(GP + EM) NPs to effectively reduce ROS levels in atherosclerotic plaques.

The accumulation of ROS within atherosclerotic plaques has been shown to exacerbate endothelial cell injury and the expression of chemokines [55]. During the early stages of atherogenesis, dysfunctional endothelial cells secrete ICAM-1 [56], a factor implicated in the disease progression. In order to investigate whether TK-MLP@(GP + EM) NPs can inhibit this process in the treatment of AS, immunofluorescence staining was performed on a cross-section of the aortic root. As shown in Fig. 7C&D, the expression level of ICAM-1 in CD31-labeled endothelial cells was significantly increased by 7.4-fold in the Model group, displaying a strong yellow fluorescence signal compared to the Control group. However, treatment with TK-MLP@(GP + EM) NPs led to a notable 53% decrease in the expression of ICAM-1 in endothelial cells at the site of the lesion. These findings suggest that TK-MLP@(GP + EM) NPs may exert their therapeutic effects on atherosclerosis by inhibiting the upregulation of ICAM-1 in endothelial cells.

To investigate the impact of TK-MLP@(GP + EM) NPs on endothelial barrier integrity further, we analyzed the localization of VE-cadherin in the aortas. In the Model group, we observed separate fluorescence signals of VE-cadherin, indicating endothelial cell dysfunction. However, after treatment with TK-MLP@(GP + EM) NPs, we observed improved continuity of fluorescence signals in endothelial cells (Fig. 7E&F). This improvement may be attributed to the elevated concentration of ROS within the plaque region, which triggers the therapeutic effect of the drugs released by the nanomaterials on endothelial repair.

These findings suggest that TK-MLP@(GP + EM) NPs have the ability to restore endothelial cell function and suppress adhesion molecule expression by reducing ROS levels in the plaque area.

### TK-MLP@(GP + EM) NPs can regulate cholesterol efflux in ApoE-/- atherosclerosis mice

**Fig. 8:**
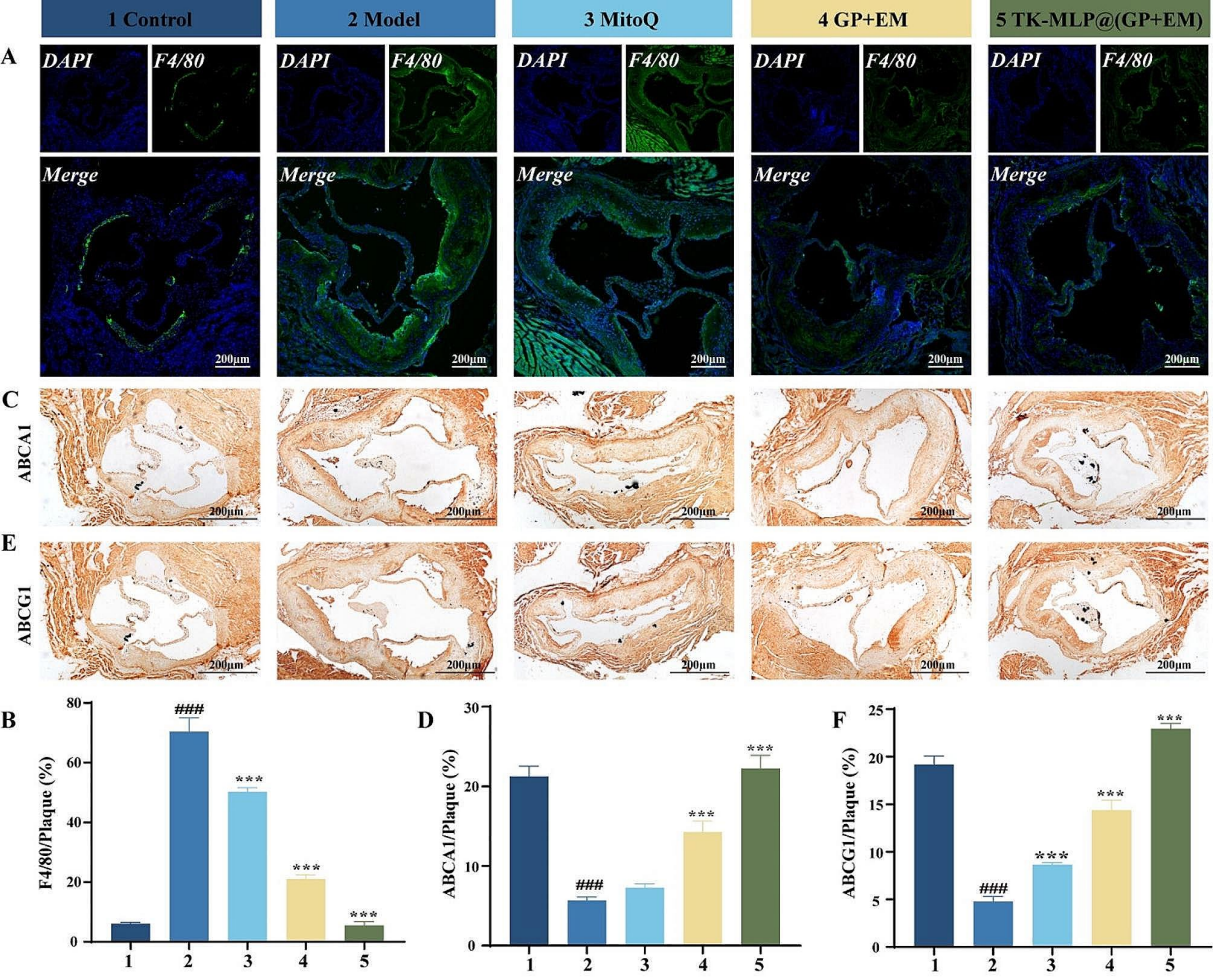
TK-MLP@(GP + EM) NPs can regulate cholesterol efflux of macrophages. (A) Representative immunofluorescence images and (B) quantitative analysis of macrophages in aortic root sections from ApoE^−/−^ mice after different treatments. (C-F) Representative histochemistry images of aortic root cross-sections stained with ABCA1 antibody and ABCG1 antibody. Scale bar = 200 μm. *n* = 3, ^###^*P* < 0.001 vs. the Control. *** *P* < 0.001 vs. the Model.

It has been reported that macrophage infiltration is a characteristic of atherosclerotic plaques [57]. In accordance with this, Fig. 7C confirmed that TK-MLP@(GP + EM) NPs effectively inhibit the production of ICAM-1 by endothelial cells. Considering this, we further investigated whether TK-MLP@(GP + EM) NPs could reduce macrophage infiltration. As shown in Fig. 8A&B, the Model group exhibited a significant amount of macrophage infiltration in the aortic root tissue sections, with a remarkable 11.5-fold increase in F4/80 expression compared to the Control group. However, the presence of macrophages was reduced to various degrees in the MitoQ group, GP + EM group, and TK-MLP@(GP + EM) NPs group, compared to the Model group. Notably, TK-MLP@(GP + EM) NPs demonstrated the most significant reduction in F4/80 expression and exhibited the most effective reduction in macrophage infiltration. These findings indicate that TK-MLP@(GP + EM) NPs can effectively reduce macrophage infiltration by inhibiting the secretion of ICAM-1 by endothelial cells.

Previous studies have demonstrated that elevated levels of ABCA1 and ABCG1 can inhibit the progression of atherosclerosis by facilitating cholesterol efflux [48]. Our immunofluorescence staining results of paraffin sections from the aortic root revealed that treatment with TK-MLP@(GP + EM) NPs led to increased expression of ABCA1 and ABCG1 (Fig. 8C-F). This indicates that TK-MLP@(GP + EM) NPs not only promote endothelial cell repair but also enhance the expression of ABCA1 and ABCG1, thereby facilitating cholesterol efflux and effectively reducing intracellular lipid accumulation. These findings highlight the potential of TK-MLP@(GP + EM) NPs in improving atherosclerosis.

Additionally, blood biochemical analysis showed that TK-MLP@(GP + EM) NPs decreased the serum levels of TC, TG, and LDL-C, although not increased the levels of HDL-C in the serum (Fig. S7A-D).

### Biosafety evaluation of TK-MLP@(GP + EM) NPs

H&E staining revealed that the Control group of mice exhibited well-defined structures in their hepatic lobules, with liver cells arranged neatly. On the other hand, ApoE^−/−^ mice fed a high-fat diet displayed round vacuoles of varying sizes in their liver cells, indicating liver steatosis. However, treatment with TK-MLP@(GP + EM) NPs significantly improved the condition of liver steatosis. Notably, there was no evidence of tissue degeneration or injury in other organs, such as the heart, spleen, lung, and kidney (Fig. S8A), suggesting that the treatment did not cause any noticeable side effects. Blood routine indexes and parameters from the Liver-kidney assay were also within the normal range in TK-MLP@(GP + EM) NP-treated mice (Fig. S8B&C). Serum levels of liver and kidney function markers in mice further confirmed the biosafety of TK-MLP@(GP + EM) NPs. Therefore, our findings demonstrate the safety and efficacy of TK-MLP@(GP + EM) NPs in treating AS.

## Conclusion

In this study, we developed a ROS-responsive biomimetic nano complex called TK-MLP@(GP + EM) NPs, a drug delivery system (DDS) capable of efficiently delivering GP and EM to endothelial cells and macrophages within the AS plaque.

Specifically, this biomimetic nanocomplexes possesses excellent biomimetic properties and a prolonged circulation time. Furthermore, it exhibits remarkable targeting capabilities, specifically accumulating within the AS plaque. Importantly, the DDS demonstrates an ideal capacity to release the drug in a microenvironment enriched with ROS. Moreover, our results showed that DDS can efficiently co-delivery of GP and EM to both endothelial cells and macrophages. The above highlight the significant potential of this nanocomplexes as a promising DDS.

In addition to the excellent function of DDS, the synergistic effect of GP and EM also plays a key role in inhibiting apoptosis of HUVECs, protecting endothelial cells from damage, and promoting cholesterol efflux from macrophages and reducing lipid accumulation, leading to the regression of AS plaque.

Through our study, we have improved the scientific knowledge surrounding the development and application of ROS-responsive biomimetic nanocomplexes for targeted AS therapy. This research paves the way for further investigations and potential clinical applications in the treatment of AS.

### Electronic supplementary material

Below is the link to the electronic supplementary material.

Supplementary Material 1

## Data Availability

No datasets were generated or analysed during the current study.
