# Supplementary material for: Synergistic dual cell therapy for atherosclerosis regression: ROS-responsive Bio-liposomes co-loaded with Geniposide and Emodin
Source: J Nanobiotechnology. 2024 Mar 25;22:129. doi: 10.1186/s12951-024-02389-5 (PMC10962080; doi:10.1186/s12951-024-02389-5)
Supplement: Supplementary file 1 — Supplementary Material 1 [file 12951_2024_2389_MOESM1_ESM.docx]

**Synergistic Dual Cell Therapy for Atherosclerosis Regression: ROS-Responsive Bio-Liposomes Co-Loaded with Geniposide and Emodin**

Zhenxian Li^a#^, Haimei Zhu^e#^, Hao Liu^c^, Dayue Liu^d^, Jianhe Liu^a^, Yi Zhang^a^, Zhang Qin^a^, Yijia Xu^a^, Yuan Peng^a^, Lihua Ruan^a^, Jintao Li^a^, Yao He^a^, Bin Liu^b⁎^, Yun Long^a⁎^

*^a^ Department of Cardiology, The First Hospital of Hunan University of Chinese Medicine, Changsha 410007, China*

*^b^ College of Biology, Hunan University, Changsha 410082, China*

^c^ *Department of Rehabilitation, The Second Xiangya Hospital, Central South University, Changsha 410011, China*

*^d^ NHC Key Laboratory of Metabolic Cardiovascular Diseases Research, Ningxia Medical University, Yinchuan 750004, China*

*^e^ Department of Pain, The First Hospital of Hunan University of Chinese Medicine, Changsha 410007, China*

⁎ Corresponding authors at:

Yun Long, Ph.D

The First Hospital of Hunan University of Chinese Medicine, Changsha 410007, China.

Bin Liu, Ph.D

College of Biology, Hunan University, Changsha 410082, China

E-mail addresses: wwlyf@126.com (Y. Long); binliu2001@hotmail.com (B. Liu).

^#^ These authors contributed equally to this work and should be regarded as the first authors.

## Fig. S1


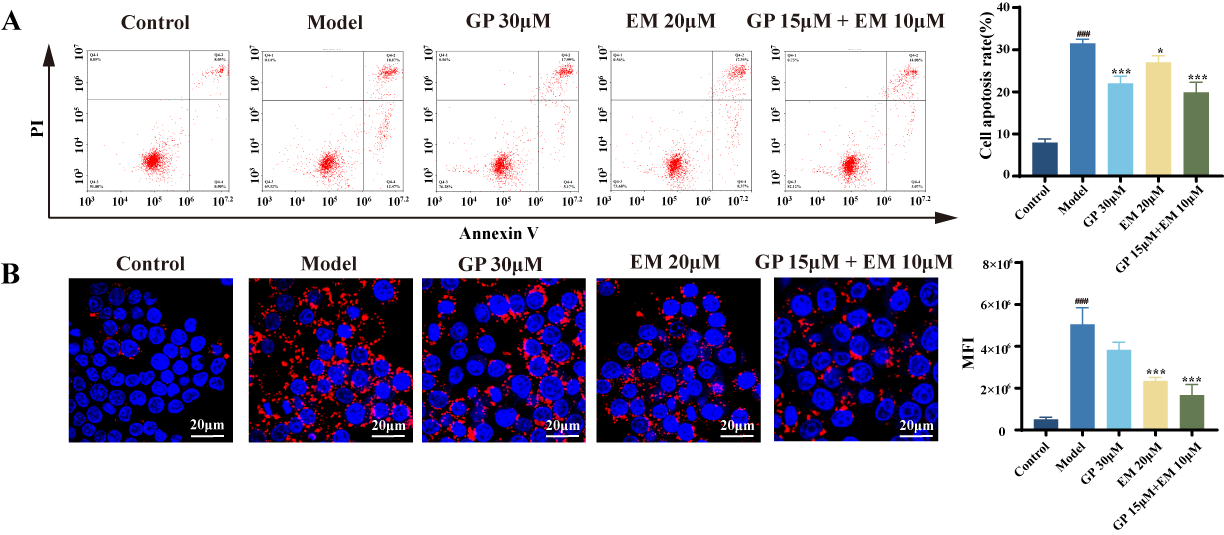


Fig. S1 (A) Apoptosis rate of HUVECs detection after treatment with GP (30μM), EM (20μM), and GP+EM (15μM +10μM) by ﬂow cytometry. (B) Confocal fluorescence images and semi-quantification analysis of cellular uptake of DiI-oxLDL in RAW 264.7 cells treated by GP (30μM), EM (20μM), and GP+EM (15μM +10μM). Scale bar = 20 μm. Scale bar = 20 μm. *n* = 3, ^###^ *P* < 0.001 *vs.* the Control. * *P* < 0.05, ** *P* < 0.01, *** *P* < 0.001 *vs.* the Model.

## Fig. S2


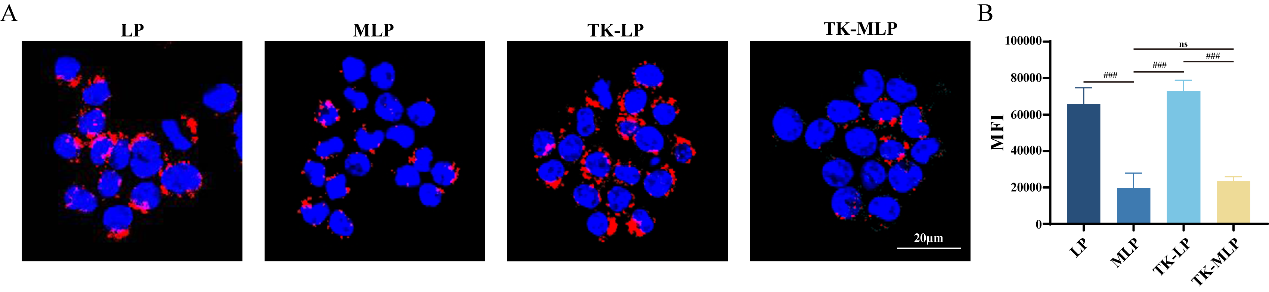


**Fig. S2** Immune-escape properties of TK-MLP@Dil NPs *in* *vitro*. (A&B) Confocal images and mean fluorescence intensity (MFI) of different NPs phagocytosed by RAW264.7 cells. Scale bars = 20 μm. *n* = 3.

## Fig. S3


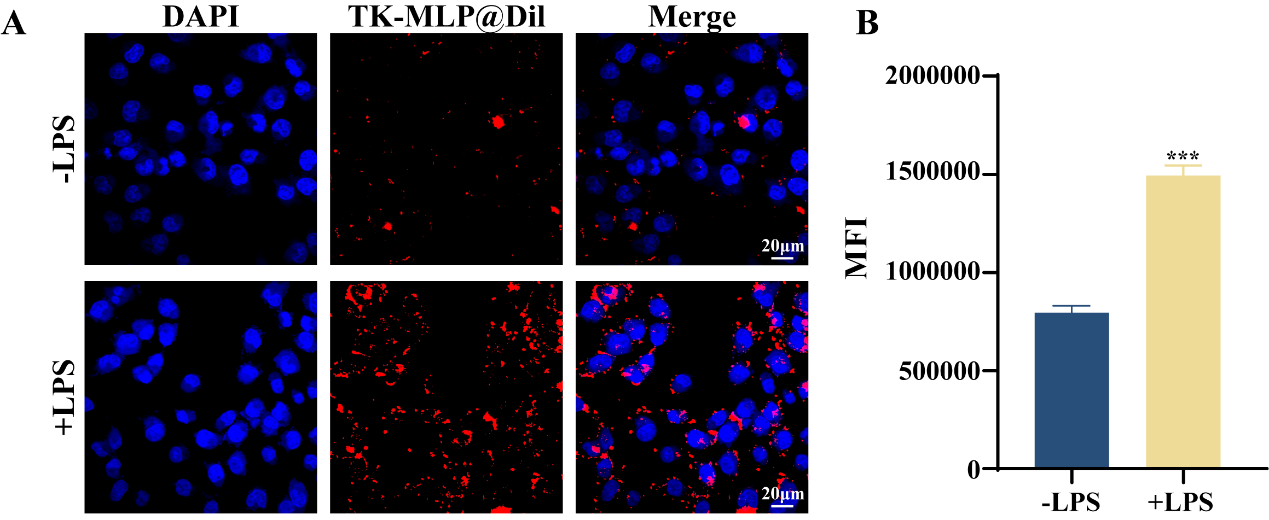


**Fig. S3** Cellular uptake and quantitative analysis of TK-MLP@Dil NPs by HUVECs treated with or without LPS. Scale bar = 20 μm*. n* = 3, ^***^*P* < 0.001.

## Fig. S4


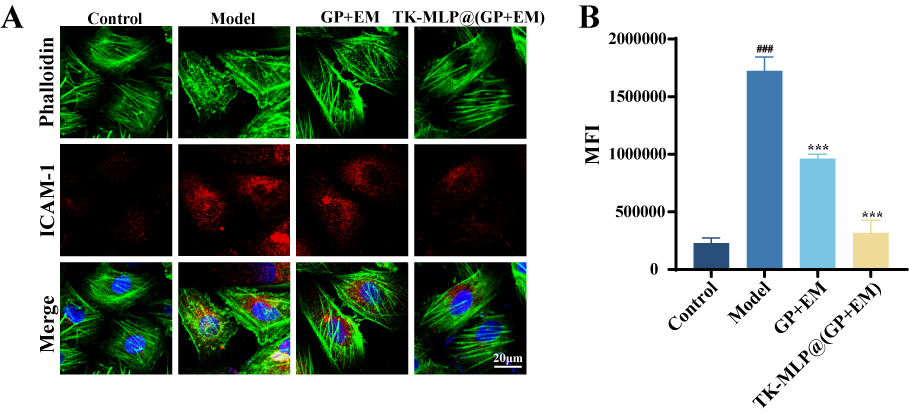


**Fig. S4** Representative confocal microscopy images of HUVECs treated with LPS stained with CoraLite® Plus 488-conjugated Phalloidin (green) and anti-ICAM-1 staining (red). Scale bar = 20 μm*. n* = 3, ^###^ *P* < 0.001 *vs.* the Control. *** *P* < 0.001 *vs.* the Model.

## Fig. S5


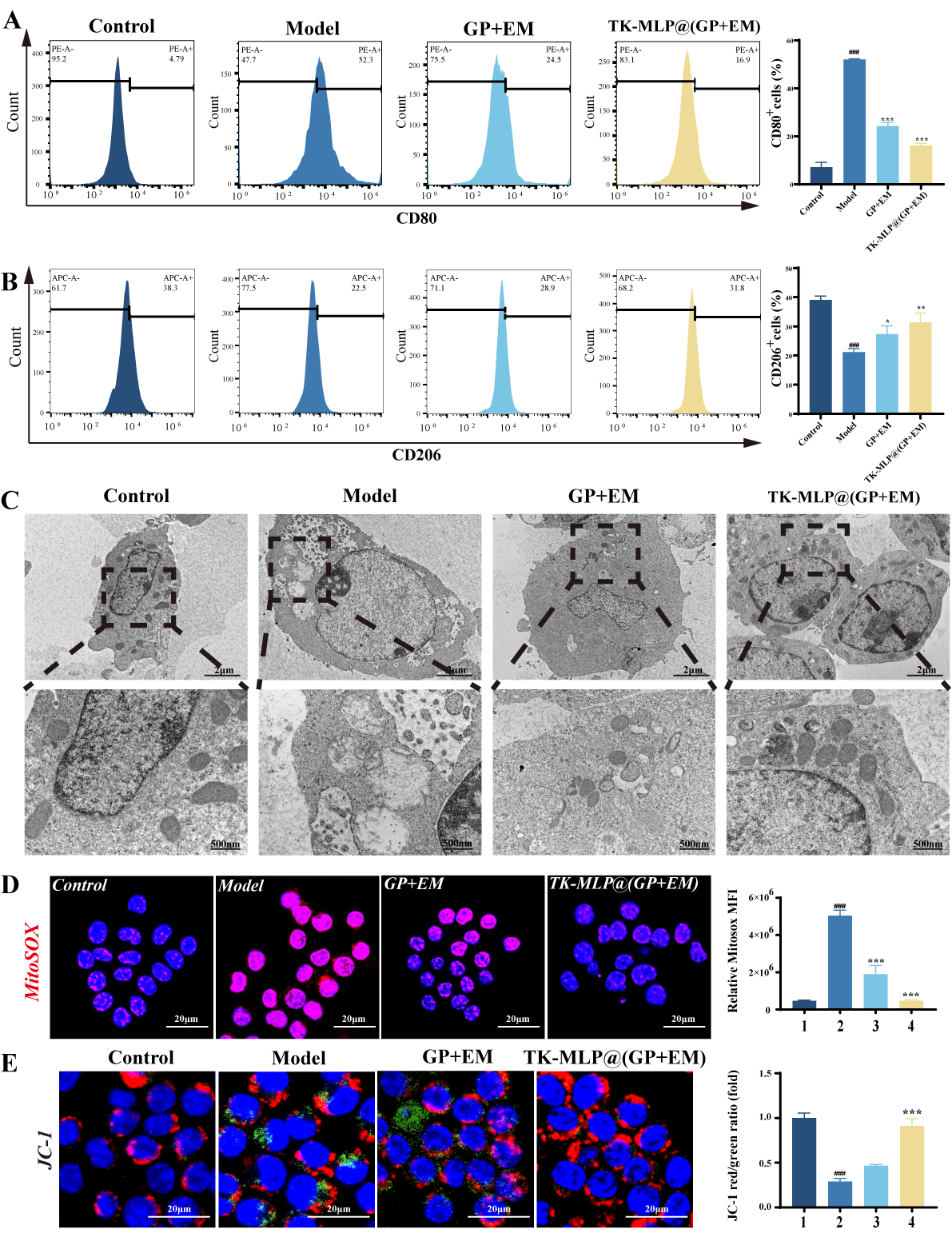


**Fig. S5** Effects of TK-MLP@(GP+EM) NPs on macrophage re-polarization and mitochondrial function during this process. (A&B) Flow cytometry profiles of M1 phenotype highly expressed CD80 and M2 phenotype highly expressed CD206. (C) The ultrastructure of mitochondria in RAW264.7 cells was observed using a transmission electron microscope (TEM). (D) Mitochondrial superoxide levels by MitoSOX Red fluorescent staining with MFI quantification. (E) Mitochondrial membrane potential by JC-1 staining with calculation of the ratio of red MFI (aggregated JC-1) to green MFI (monomer JC-1). Scale bar = 20 μm*. n* = 3, ^###^ *P* < 0.001 *vs.* the Control. **P* < 0.05, ** *P* < 0.01, *** *P* < 0.001 *vs.* the Model.

## Fig. S6


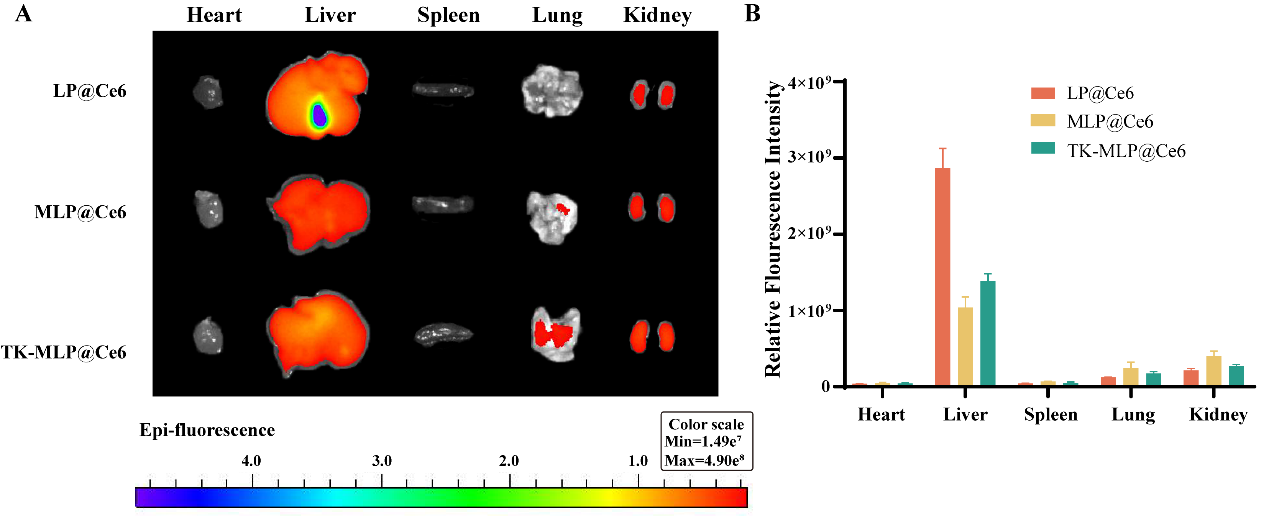


**Fig. S6** Distribution of different NPs in major organs of ApoE^-/-^ mice. (A) Fluorescence imaging of the major organs of ApoE^-/-^ mice with different treatments for 12 h. (B) Relative fluorescence signal of major organs. *n* = 3.

## Fig. S7


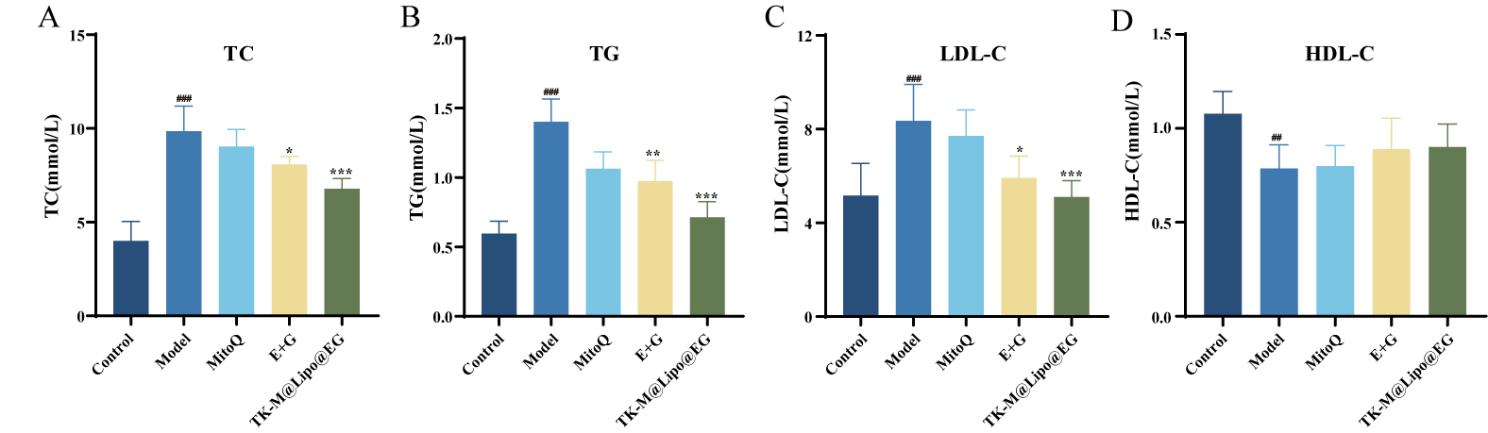


**Fig.S7** (A-D) TC, TG, LDL-C, and HDL-C were detected in the serum of ApoE^-/-^ mice. *n* = 6, ^##^ *P* < 0.01, ^###^ *P* < 0.001 *vs.* the Control. * *P* < 0.05, ** *P* < 0.01, *** *P* < 0.001 *vs.* the Model.

## Fig. S8


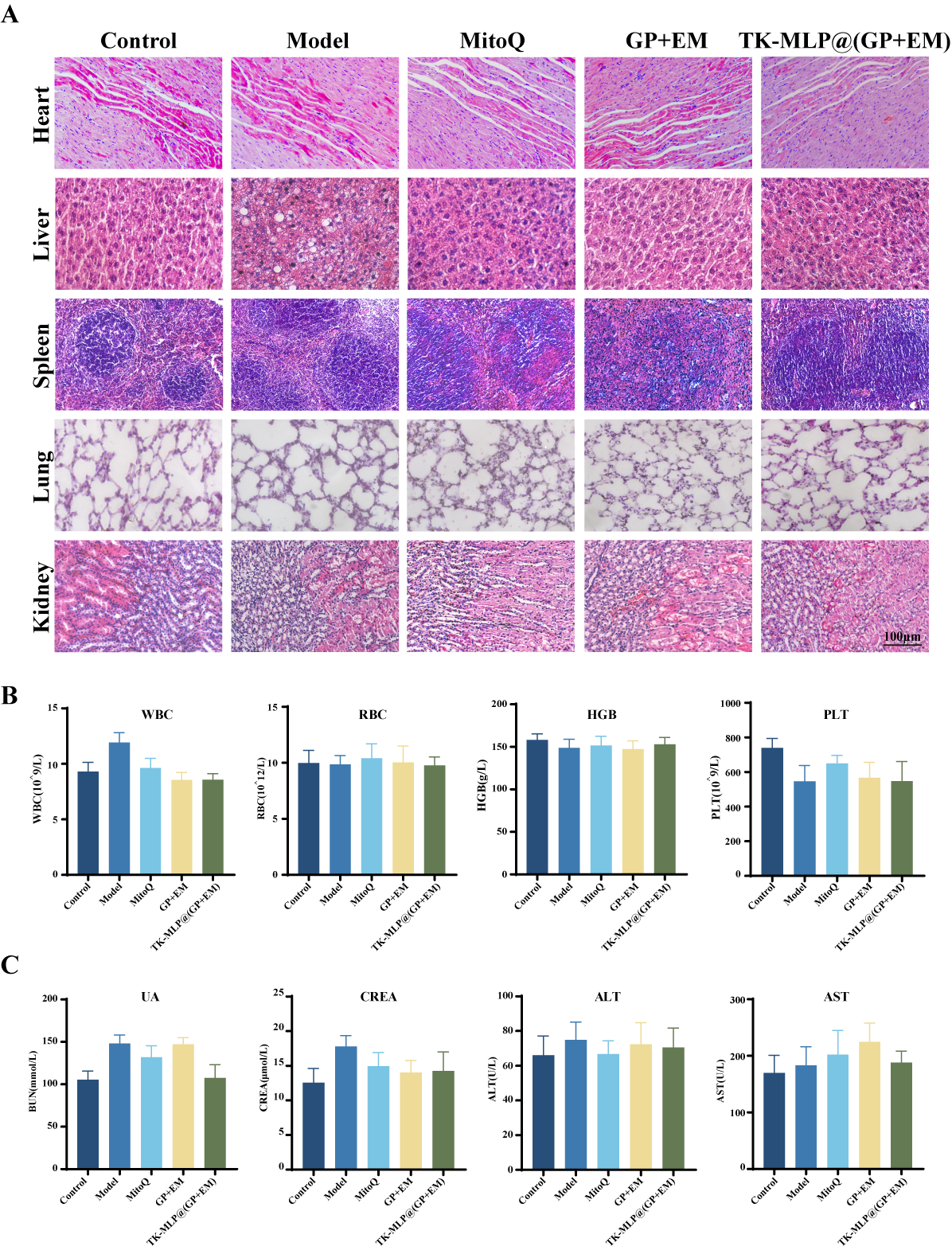


Fig.S8 (A) H&E-stained sections of heart, liver, spleen, lung and kidney of ApoE^-/-^ mice with different treatments. *n* = 6. (B&C) Blood routine and liver-kidney assays. *n* = 6.
